# Myristoyl group-aided protein import into the mitochondrial intermembrane space

**DOI:** 10.1038/s41598-018-38016-1

**Published:** 2019-02-04

**Authors:** Eri Ueda, Yasushi Tamura, Haruka Sakaue, Shin Kawano, Chika Kakuta, Shunsuke Matsumoto, Toshiya Endo

**Affiliations:** 1 Department of Chemistry, Graduate School of Science, Nagoya University, Chikusa-ku, Nagoya 464-8602 Japan; 2 Department of Material and Biological Chemistry, Faculty of Science, Yamagata University, 1-4-12 Kojirakawa-machi, Yamagata, 990-8560 Japan; 3 Faculty of Life Sciences, Kyoto Sangyo University, Kamigamo-motoyama, Kita-ku, Kyoto 603-8555 Japan; 4 Research Center for Protein Dynamics, Kyoto Sangyo University, Kamigamo-motoyama, Kita-ku, Kyoto 603-8555 Japan; 5 Present Address: Department of Biological Science, Graduate School of Science, Osaka University, 1-1 machikaneyama-cho, Toyonaka, 560-0043 Osaka Japan

## Abstract

The MICOS complex mediates formation of the crista junctions in mitochondria. Here we analyzed the mitochondrial import pathways for the six yeast MICOS subunits as a step toward understanding of the assembly mechanisms of the MICOS complex. Mic10, Mic12, Mic26, Mic27, and Mic60 used the presequence pathway to reach the intermembrane space (IMS). In contrast, Mic19 took the TIM40/MIA pathway, through its CHCH domain, to reach the IMS. Unlike canonical TIM40/MIA substrates, presence of the N-terminal unfolded DUF domain impaired the import efficiency of Mic19, yet N-terminal myristoylation of Mic19 circumvented this effect. The myristoyl group of Mic19 binds to Tom20 of the TOM complex as well as the outer membrane, which may lead to “entropy pushing” of the DUF domain followed by the CHCH domain of Mic19 into the import channel, thereby achieving efficient import.

## Introduction

Mitochondria are essential organelles in eukaryotic cells that mediate energy generation, production of metabolites, and regulation of apoptosis. Mitochondria consist of two membranes, the outer membrane (OM) and inner membrane (IM), and two aqueous compartments, the intermembrane space (IMS) and matrix. While the OM functions as an envelope of the organelle, it mediates the exchange of small soluble molecules with the cytosol through porin and for the exchange of insoluble metabolites like lipids with other organelles such as the endoplasmic reticulum (ER) and vacuoles through interorganelle membrane contacts^1,2^. The IM consists of two distinct regions, the inner boundary membrane (IBM) and crista membrane^3–5^. The IBM is a planner IM region that runs parallel to the OM^3^. Cristae are tubular or lamellar membrane invaginations of the IM, which are connected to the IBM by narrow constrictions called crista junctions (CJs)^3^. CJs are narrow constrictions that connect the IMS with the intracrista space, but probably pose a diffusion barrier for metabolites, soluble proteins and membrane proteins between the IMS plus IBM and the intracrista space plus crista membrane^6–8^. Since mitochondrial cristae and oxidative phosphorylation functions are directly connected, formation of cristae structures have an impact on cellular metabolism through mitochondrial bioenergetics. Cristae formation requires dimerization of the F_1_F_o_-ATP synthase, which generates a significant curvature of the IM for forming a tip of the cristae^9,10^, and the presence of the mitochondrial cristae organizing system (MICOS) complex, which mediates formation of the CJs with a negative curvature and contacts between the IM and OM^11–14^. Recent studies showed that formation of lamellar cristae further depends on the IM fusion protein Mgm1 while tubular cristae are formed by invaginations of the IBM independently of Mgm1^15^.

**Figure 1:**
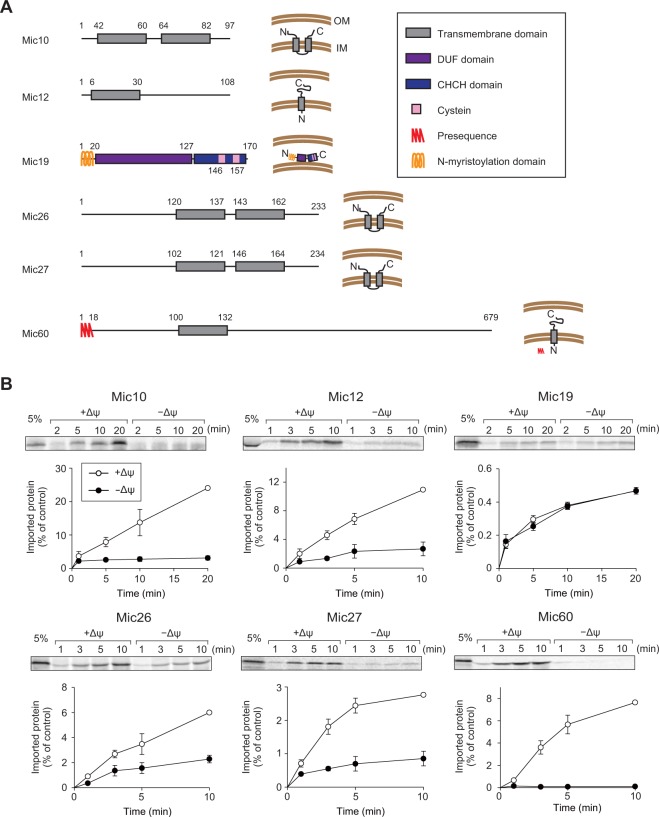
Import of MICOS subunits except for Mic19 requires ΔΨ. (**A**) Schematic diagrams of the amino-acid sequences (left) and membrane topologies (right) of yeast MICOS subunits. Mic19 is a peripheral IM protein, and the other MICOS subunits are integral membrane proteins. (**B**) The indicated radiolabeled proteins were incubated with mitochondria with ΔΨ (open circles) or without ΔΨ (closed circles) for the indicated times at 25 °C. After proteinase K (PK) treatment, mitochondria were subjected to SDS-PAGE and radioimaging. Imported, protease-protected proteins were quantified, and the amounts of the radiolabeled proteins added to each reaction were set to 100%. Values are mean ± SEM (*n = *3). Full-length gel images are presented in Supplementary Fig. S4.

The MICOS complex is an evolutionary conserved IM protein complex, which consists of at least six subunits in yeast, Mic10, Mic12, Mic19, Mic26, Mic27, and Mic60^16,17^. The mammalian MICOS complex further contains Mic25, a Mic19 homolog, and several interacting partners^16,17^. Apparently the MICOS complex is assembled from two distinct sub-complexes^18–20^. The Mic10 sub-complex consists of integral membrane proteins with one or two transmembrane (TM) segments, Mic10, Mic12, Mic26, and Mic27, and the Mic60 sub-complex contains an integral membrane protein with a single N-terminal TM segment, Mic60, and a peripheral membrane protein Mic19 (plus a Mic19 homolog Mic25 in mammals)^18–20^ (Fig. 1). Mic10 of the Mic10 sub-complex oligomerizes on its own, thereby bending the IM, and a subpopulation of Mic10 molecules also associate with the dimeric form of ATP synthase, thereby contributing to crista rim formation^21^. The IMS domain of Mic60 functions as a platform for interactions with OM proteins including the TOM and TOB/SAM complex proteins, thereby transiently forming contacts between the OM and IM. Mic19 was found to associate with cytochrome oxidase subunit IV (CoxIV), as well^22^. However, precise mechanisms of how each MICOS sub-complex is made from their constituent proteins and how the two sub-complexes assemble together to form CJ structures are largely unclear.

In the present study, we analyzed the import pathways for the six yeast MICOS subunits as a first step toward understanding of the assembly mechanisms of the MICOS complex. In contrast to the other five subunits, which used the presequence import pathway involving the TOM complex in the OM and TIM23 complex in the IM, import of Mic19 required the TOM complex and Tim40/Mia40^23,24^, which mediates oxidative folding in the IMS^25–27^. Besides, the Mic19 import depended partly on the previously uncharacterized pathway that requires the myristoyl group attached to the N-terminal domain^23,28^. We will discuss how N-myristoylation contributes to the efficient import of Mic19 into mitochondria.

## Results and Discussion

### Import of the MICOS subunits other than Mic19 is mediated by the TIM23 complex

The five yeast MICOS subunits, Mic10, Mic12, Mic26, Mic27, and Mic60, are integral IM proteins with one or two TM segments while Mic19 is a peripheral IM protein on the IMS side (Fig. 1A)^11–13^. Except for presequence-containing Mic60, import pathways for the other 5 MICOS subunits are not obvious from their amino-acid sequences as they lack presequences (Fig. 1A). We thus performed *in vitro* import of those proteins into mitochondria in the presence or absence of the membrane potential across the IM (ΔΨ). Mic60 was efficiently imported into the protease-protected location of mitochondria with ΔΨ, but not at all in the absence of ΔΨ, as expected for the presequence-pathway import (Fig. 1B). Mic60 import required Tim50 of the TIM23 complex (Fig. S1), but not the mitochondrial Hsp70 import motor, Ssc1 (Fig. S2A). Since Mic60 contains a TM segment starting from 82-residue downstream to the presequence cleavage site, the TM segment is probably released laterally into the IM by the stop-transfer mechanism^29^. It is to be noted that presequence-containing proteins for lateral release from the TIM23 complex may not require Ssc1 if a TM segment is close to the presequence-cleavage site and the following C-terminal domain does not fold tightly^30^.

Import of Mic10, Mic12, Mic26, and Mic27 was significantly hampered by dissipation of ΔΨ, yet there remained slight residual import into the PK-protected location even in the absence of ΔΨ. Similar ΔΨ-independent import of the TIM23 pathway substrates was reported for a small single-spanning IM protein like a subunit of the F_1_F_o_-ATPase, Su-e^31^. Import of Mic10, Mic12, Mic26, and Mic27 required Tim50 of the TIM23 complex (Fig. S1), but not the import motor Ssc1 (Fig. S2A). Since Mic12 spans the IM once with the N^in^-C^out^ topology (Fig. 1A), it is probably inserted into the IM via the TIM23 complex by the stop-transfer mechanisms. On the other hand, Mic10, Mic26, and Mic27 span the IM twice and take N^out^-C^out^ topology with a loop between the first and second TM segments exposed to the matrix (Fig. 1A). A possible mechanism to achieve the membrane topology of these proteins would be that their first TM segment reaches the matrix completely through the TIM23 complex and then inserts into the IM from the matrix side by Oxa1^32^, while the second TM segment being released laterally into the IM from the TIM23 complex by the stop-transfer mechanism^33^, although this possibility needs to be experimentally tested. On the other hand, import of Mic19 was not affected by the absence of ΔΨ at all or by functional defects of Tim50 (Fig. S1) or Ssc1 (Fig. S2A).

### Mic19 import depends on Tim40/Mia40 and the presequence receptor Tom20, but not Tom70/71

**Figure 2:**
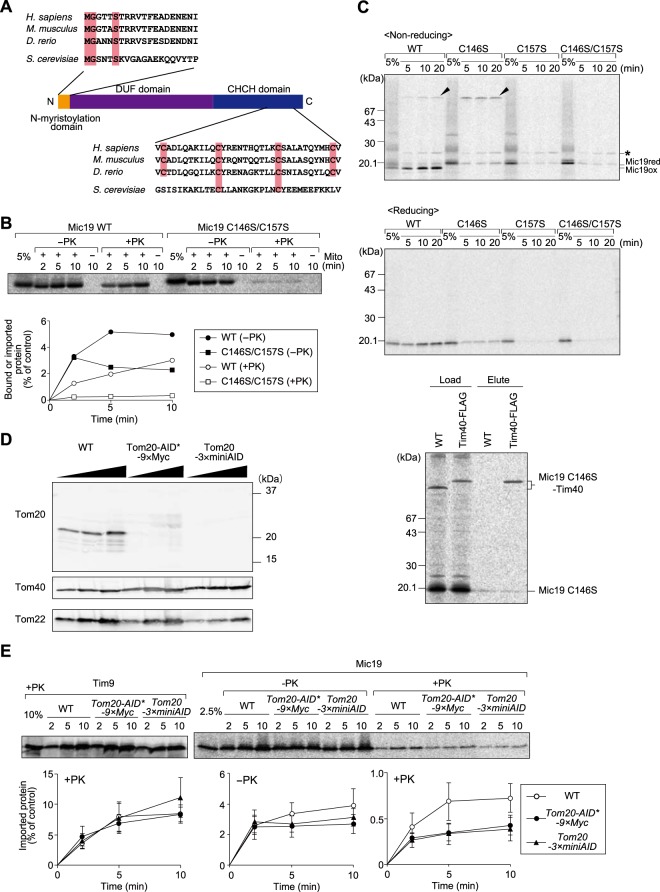
Mic19 import requires the CX_10_C motif and Tom20. (**A**) Amino-acid sequence alignment of the N-myristoylation domain and CHCH domain of Mic19 from *Homo sapiens*, *Mus musculus*, *Danio rerio*, and *Saccharomyces cerevisiae*. (**B**) Radiolabeled wild-type (WT) Mic19 and its C146S/C157S mutant were incubated with mitochondria for the indicated times at 25 °C. After PK treatment, mitochondria were subjected to SDS-PAGE and radioimaging. Bound proteins (−PK) and imported, protease-protected proteins (+PK) were quantified, and the amounts of the radiolabeled proteins added to each reaction were set to 100%. (**C**) Radiolabeled wild-type (WT) Mic19 and its mutants (C146S, C157S and C146S/C157S) were incubated with mitochondria for the indicated times at 25 °C. Then the mitochondria were treated with 50 μg/ml PK and 50 mM IAA (2-iodoacetamide) for 20 min on ice and subjected to SDS-PAGE with 5 M urea in the absence (non-reducing) or presence (reducing) of 5% β-mercaptoethanol, and radioimaging. Arrowheads and asterisk indicate Mic19-Tim40 conjugates and nonspecific signals, respectively. In the lowermost panel, we incubated RI-labeled Mic19-C146S with mitochondria containing WT Tim40 or Tim40-FLAG for 20 min at 25 °C. After treatment with 50 mM IAA, the mitochondria were solubilized with 1% digitonin, and Tim40 conjugates were isolated by affinity purification for the FLAG tag. 5% of the applied sample (Load) and 100% of the eluted fraction (Elute) were analyzed by SDS-PAGE and radioimaging. Band shifts by the attached FLAG tag and pull-down by the anti-FLAG antibody confirmed that the 75 kD proteins (indicated with arrowheads in the uppermost panel), which were derived from WT Mic19 or Mic19C146S, contained Tim40 and thus represented Mic19-Tim40 conjugates. (**D**) The indicated proteins were analyzed by SDS-PAGE followed by immunoblotting for the wild-type strain (WT) and those with Tom20-AID*-9* × *Myc and Tom20-3* × *miniAID instead of Tom20 after cultivation in lactate medium (+0.05% glucose) at 30 °C. (**E**) The indicated radiolabeled proteins were incubated with the indicated mitochondria for the indicated times at 25 °C. After treatment with or without PK (50 μg/ml) for 20 min on ice, mitochondria were subjected to SDS-PAGE and radioimaging. Bound proteins (-PK) and imported, protease-protected proteins (+PK) were quantified, and the amounts of the radiolabeled proteins added to each reaction were set to 100%. Values are mean ± SEM (*n = *3). Full-length blot/gel images are presented in Supplementary Fig. S5.

Tim40/Mia40 and Erv1 mediate import and disulfide bond formation of soluble proteins with a twin CX_3_C or CX_9_C motif in the IMS without requiring ΔΨ (TIM40/MIA pathway)^8,34–36^. Previous studies reported that human Mic19 (ChChd3)^37^ and yeast Mic19 are imported into mitochondrial IMS via the TIM40/MIA pathway. Mic19 proteins from various organisms consist of the N-terminal segment containing a myristoylation motif followed by a domain with an unknown function called a DUF (domain of unknown function) domain and the C-terminal CHCH (coiled-coil helix coiled-coil helix) domain, which often contains a twin CX_9_C motif (Fig. 2A)^28^. Although fungal Mic19 proteins including yeast Mic19 do not have a typical twin CX_9_C motif in the CHCH domain, they instead have a CX_10_C motif, which was shown to be important for its import^24^. Indeed, the C146S/C157S mutation in the CX_10_C motif of yeast Mic19 impaired its import into the IMS (Fig. 2B). Under non-reducing conditions, Mic19C146S, not Mic19C157S or Mic19C146S/C157S, generated a 75kD band (Fig. 2C, uppermost panel), which was confirmed to be a mixed-disulfide intermediate with Tim40/Mia40 (Fig. 2C, lowermost panel) through C298 of Tim40/Mia40^38^. A mix-disulfide intermediate was slightly observed for even wild-type Mic19, which mainly formed an oxidized form upon its import (Fig. 2C). Our results thus strongly support the model that the TIM40/MIA pathway is the primary import pathway for Mic19 and that Mic19 forms a mix-disulfide intermediate with Tim40 through the second cysteine residue, Cys157.

Substrates for the TIM40/MIA pathway usually do not depend on either the presequence receptors Tom20 and Tom22^39^ or receptors Tom70/71 for presequence-less proteins. We thus analyzed if *in vitro* import of Mic19 depends on the mitochondrial surface receptors. *In vitro* import of Mic19, like presequence-containing pSu9-DHFR but unlike presequence-less ADP-ATP carrier (AAC), did not depend on the receptors for presequence-less proteins, Tom70 and Tom71 (Fig. S2B,C). We then tested involvement of the presequence-receptor Tom20 in the Mic19 import. For this purpose, we tried to delete the Tom20 receptor domain by an auxin-based degron (AID) system *in vivo*^40^. However, we found that introduction of the AID tag or 3 × miniAID tag to Tom20 destabilized Tom20 even in the absence of auxin, leaving the partner receptor Tom22 level less significantly affected (Fig. 2D). We thus isolated mitochondria with a reduced level of Tom20 from the strains expressing Tom20 with the AID tag or 3 × miniAID tag and performed *in vitro* import. Surprisingly, import of Mic19 was retarded in mitochondria lacking Tom20 while that of the canonical TIM40/MIA substrate Tim9 was not affected (Fig. 2E).

### N-myristoylation enhances import of Mic19

**Figure 3:**
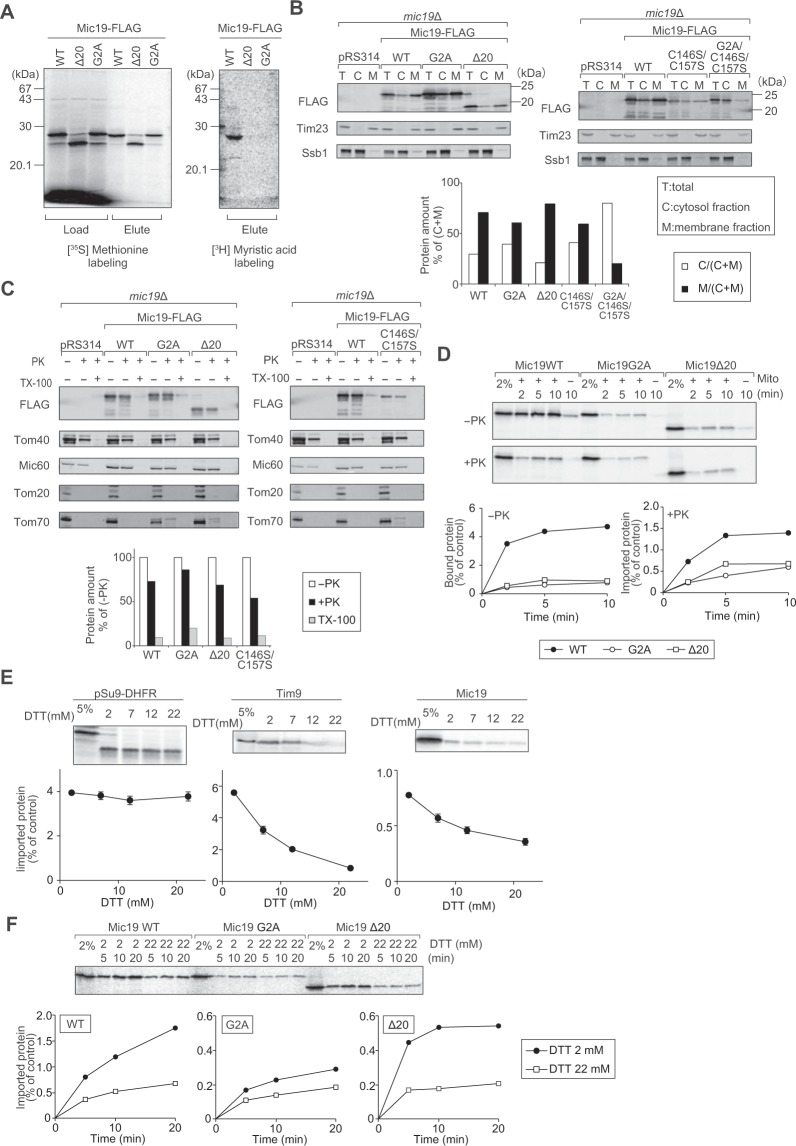
Mic19 receives N-myristoylation, which is essential for efficient import. (**A**) Mic19 with the 3 × FLAG tag (Mic19-FLAG, WT) and its myristoylation domain mutants were translated with reticulocyte lysate in the presence of [^35^S]-methionine or [^3^H]-myristic acid plus cold methionine. The translated proteins (Load) were affinity purified for the FLAG tag (Elute), and analyzed by SDS-PAGE and radioimaging. (**B**) *mic19*Δ cells expressing Mic19-FLAG (WT) or its myristoylation variants from the pRS314 plasmid and their vector control cells (pRS314) were subjected to cell fractionation. T, total; M, membrane fractions; C, soluble cytosolic fractions. Proteins were analyzed by SDS-PAGE and immunoblotting with the indicated antibodies. Total amounts (M + C) were set to 100%. Tim23 is a mitochondrial membrane protein and Ssb1 is a cytosol protein. (**C**) Mitochondria were isolated from the *mic19*Δ cell expressing Mic19-FLAG (WT) or its myristoylation variants from the pRS314 plasmid and their vector control cells (pRS314) after cultivation in SCLac medium and subjected to treatment with or without 50 μg/ml PK and/or 1% Triton X-100 for 20 min on ice. Proteins were analyzed by SDS-PAGE and immunoblotting with the indicated antibodies. The amounts without PK and Triton X-100 treatment were set to 100%. (**D**) Radiolabeled Mic19 (closed circles) and its myristoylation domain mutants (open circles and squares) were incubated with mitochondria for the indicated times at 25 °C. Bound and imported proteins were analyzed as in Fig. 2E. (**E**) The indicated radiolabeled proteins were incubated with mitochondria in the presence of different concentrations of dithiothreitol (DTT) for the indicated times at 25 °C. As a control, 2 mM DTT was added to the reaction to prevent disulfide formation of the substrate proteins outside mitochondria. Imported proteins were analyzed as in Fig. 2E. (**F**) Radiolabeled Mic19 (Mic19WT) and its N-myristoylation motif mutants (Mic19G2A, Mic19Δ20) were incubated with mitochondria for the indicated times at 25 °C in the presence of different concentrations of DTT. Imported proteins were analyzed as in Fig. 2E. Full-length blot/gel images are presented in Supplementary Fig. S6.

Unusual dependence of Mic19 import on the presequence-receptor Tom20 suggests that Mic19 may utilize not only the pathway aided by Tim40/Mia40, but also another minor pathway aided by Tom20 for its import. In connection to this, a previous study on ChChd3, a mammalian homolog of Mic19, suggested that N-terminal myristoylation at Gly2 is important for its localization to mitochondria under the microscope *in vivo*^28^. Since yeast Mic19 also contains a myristoylation motif, MGX_3_S (X stands for any amino acid residue) at the N-terminus (Fig. 2A)^41^, we reasoned that myristoylation at Gly2 of Mic19 plays a role of, likely Tim40/Mia40 independent import of Mic19. We thus first tested if yeast Mic19 is N*-*myristoylated. We synthesized FLAG-tagged Mic19 (Mic19-FLAG), Mic19-FLAG without the myristoylation motif or lacking the N-terminal 20 residues (Mic19Δ20-FLAG) and Mic19-FLAG with a mutated myristoylation motif (Mic19G2A-FLAG) *in vitro* by using reticulocyte lysate in the presence of either [^35^S]-methionine or [^3^H]-myristic acid. While Mic19-FLAG, Mic19Δ20-FLAG, and Mic19G2A-FLAG were efficiently synthesized *in vitro*, only Mic19-FLAG, not Mic19Δ20-FLAG or Mic19G2A-FLAG, received myristoylation with [^3^H]-myristic acid (Fig. 3A). Yeast Mic19 is therefore N-myristoylated through its N-terminal myristoylation motif in reticulocyte lysate *in vitro*.

We then analyzed the effects of defective N-myristoylation and/or Cys mutations in the CX_10_C motif of Mic19 on its cellular localization (Fig. 3B). Although a defective N-myristoylation (G2A or Δ20) or mutation in the CX_10_C motif (C146S/C157S) alone did not deteriorate the membrane localization of Mic19, their combination (G2A/C146S/C157S) significantly impaired the localization of Mic19 to membranes *in vivo* (Fig. 3B). These membrane-localized Mic19 derivatives were resistant to externally added proteinase K, indicating their localization within mitochondria (Fig. 3C). Therefore N-myristoylation and the CX_10_C motif function redundantly in mitochondrial targeting of yeast Mic19 *in vivo*.

Next, we tested the role of N-myristoylation of Mic19 in mitochondrial import *in vitro* (Fig. 3D). Binding of Mic19 to mitochondria was markedly impaired by inhibition of N-myristoylation by the G2A or Δ20 mutation of Mic19 while import into mitochondria was mildly affected by these mutations. We thus tested the effects of DTT, which impairs the TIM40/MIA pathway import (Fig. 3E), on import of Mic19G2A and Mic19Δ20 *in vitro*. Although import of Mic19 was significantly impaired by the G2A or Δ20 mutation, the residual import of Mic19G2A and Mic19Δ20 was still sensitive to 22 mM DTT, like wild-type Mic19 (Fig. 3F), suggesting that N-myristoylation and the CX_10_C motif function independently in the import of Mic19.

### Myristoylation circumvents the import-impairing effect by the DUF domain

**Figure 4:**
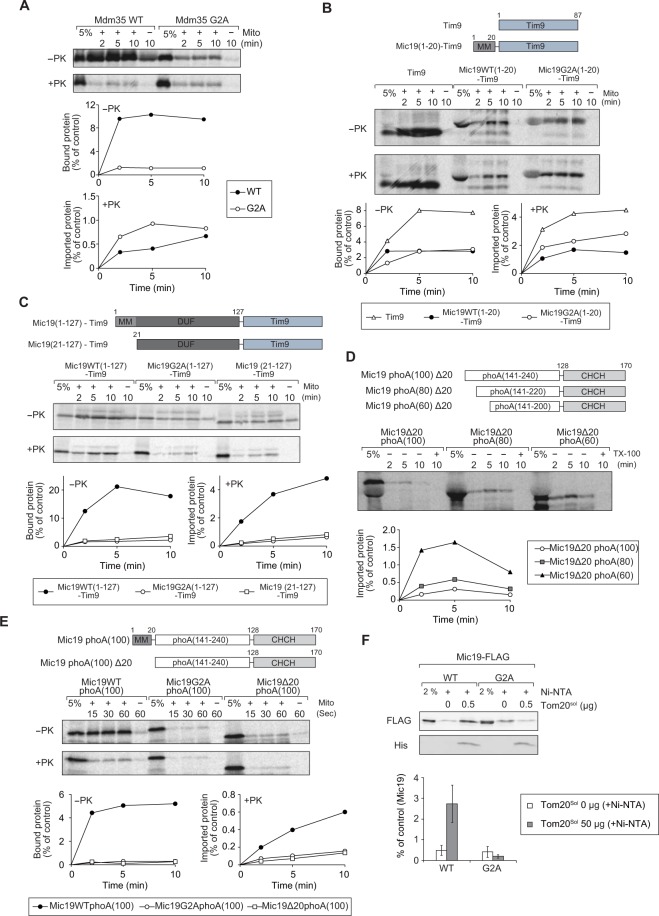
DUF domain hampers import of TIM40/MIA pathway substrates, which is circumvented by N-myristoylation. (**A–E**) The indicated radiolabeled proteins were incubated with mitochondria for the indicated times at 25 °C. Bound and imported proteins were analyzed as in Fig. 2E. (**F**) Mitochondria with Mic19WT-FLAG or Mic19G2A-FLAG were solubilized with 1% digitonin and incubated with 0.5μg of the purified cytosolic soluble domain of rat Tom20 (Tom20^sol^) bound to Ni-NTA resin or with Ni-NTA without bound Tom20^sol^ as a control. The bound proteins were eluted with 500 mM imidazole and subjected to SDS-PAGE and immunoblotting with the indicated antibodies. The amounts of Mic19WT-FLAG or Mic19G2A-FLAG added to each reaction were set to 100%. Values are mean ± SEM (*n* = 3). Full-length gel images are presented in Supplementary Fig. S7.

We next tested the effects of N-myristoylation on the import of canonical TIM40/MIA substrates. While a TIM40/MIA substrate Mdm35^42–44^ has the N-terminal myristoylation motif as well as a twin CX_9_C motif, Tim9 has only the twin CX_9_C motif, not the myristoylation motif. Although binding of Mdm35 to mitochondria was significantly reduced by the G2A mutation, its import was not affected by the G2A mutation (Fig. 4A). We then compared Tim9 and the fusion protein consisting of the N-terminal 20-residue segment of Mic19 (Mic19(1-20)), with or without the G2A mutation, followed by Tim9 for their binding to and import into isolated mitochondria (Fig. 4B). The Mic19(1-20) segment, irrespective of the presence of the myristoylation motif, did not enhance mitochondrial binding or import of Tim9, or rather partially impaired binding and import of Tim9.

Mic19, Mdm35, and Tim9 are all TIM40/MIA pathway substrates, but why does the N-terminal myristoylation enhance import of only Mic19, not Mdm35 or Tim9? We noted that Mic19 possesses a ~100-residue DUF domain between the N-terminal myristoylation domain and the CHCH domain. We attached the DUF domain i.e. residues 21–127 of Mic19 to the N-terminus of Tim9 (Mic19(21-127)-Tim9), and tested its import into mitochondria (Fig. 4C). Mic19(21-127)-Tim9 was hardly imported into mitochondria, suggesting that the N-terminal attachment of the DUF domain inhibited import of Tim9. On the other hand, when residues 1–21 containing the myristoylation motif were further attached N-terminally to Mic19(21-127)-Tim9, the resultant Mic19(1-127)-Tim9 was efficiently bound to and imported into mitochondria. However G2A mutation impaired the import ability of Mic19(1-127)-Tim9. These results suggest that the myristoylation motif is important for the import ability of the DUF-domain containing TIM40/MIA substrates.

Why does the DUF domain suppress import of TIM40/MIA substrates? Since the DUF domain is predicted by PrDOS^45^ to lack regular secondary structures, we reasoned that the presence of a long unfolded segment in front of the CHCH domain could impair its import. Indeed, when we performed limited digestion of Mic19 with the C-terminally attached FLAG tag, which was solubilized with Triton X-100 from mitochondria, with different concentrations of proteinase K (PK), the DUF domain appeared more sensitive to PK digestion than the C-terminal CHCH domain (Fig. S3). We thus attached various lengths of the unrelated unfolded segment of PhoA^46^ at the N-terminus of the Mic19 CHCH domain and tested their import (Fig. 4D). The presence of an increasing length of the PhoA segment retarded import of the Mic19 CHCH domain and made the imported proteins unstable within mitochondria. However, attachment of the Mic19(1-20) segment with the myristoylation motif, but not the one with the G2A mutation, enhanced mitochondrial binding and import of the CHCH domain with the 100-residue PhoA segment (Fig. 4E). Therefore, the N-terminal myristoylation motif of Mic19 is important for the import of the CHCH domain via the TIM40/MIA pathway in the presence of an N-terminal long unfolded segment.

What is the target of the N-terminal myristoylated segment of Mic19 in its import? Since Mic19 was, unlike conventional TIM40/MIA pathway substrates, recognized by the presequence receptor Tom20, Tom20 could be a candidate for the factor that interacts with the myristoyl group of Mic19. We thus tested binding of myristoylated Mic19 with the isolated receptor domain (residues 51–145) of rat Tom20 (Tom20^sol^)^47^ (Fig. 4F). After incubation of Mic19 and Tom20^sol^ with the C-terminal His_6_ tag, affinity pull-down for the His_6_ tag revealed specific binding of Mic19 to Tom20^sol^. The G2A mutation in the myristoylation motif of Mic19 inhibited its binding to Tom20^sol^, suggesting that the myristoyl group of Mic19 is directly recognized by Tom20. The myristoyl group of Mic19 was previously shown to interact with Tob55/Sam50 inside mitochondria^23,48^, but besides, our results here suggest that the myristoyl group of Mic19 is recognized by the cytosolic receptor domain of Tom20. Since Tom20 recognizes the hydrophobic side of the amphiphilic mitochondrial targeting signal like presequences through its hydrophobic groove^47^, the hydrophobic myristoyl group of Mic19 may well bind to the hydrophobic groove of Tom20.

### Entropic pushing model for the import of Mic19

**Figure 5:**
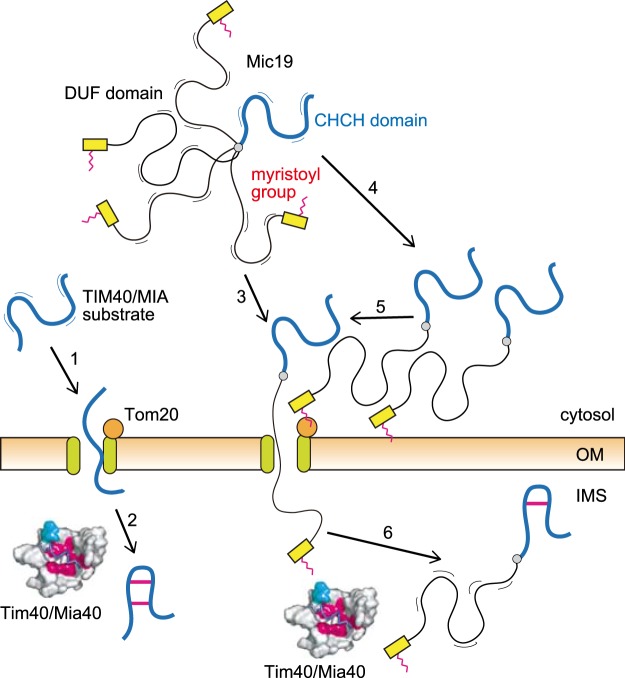
Model for the import of Mic19. Entropic pushing model for the import of Mic19 into mitochondrial IMS in comparison with the import of canonical TIM40/MIA substrates.

Why does the attachment of a DUF domain N-terminally to the CHCH domain decrease the import efficiency of Mic19, and how does the N-terminal myristoylation counteract this effect? Generally, entry of a reduced TIM40/MIA substrate domain, which is not tightly folded in the cytosol, into a narrow Tom40 import channel is a thermodynamically unfavorable process because it will decrease the conformational entropy of the substrate due to the increased excluded-volume constraint between the substrate polypeptide and the import channel (Fig. 5, step 1). This entropy decrease can be circumvented by weak binding of the substrate polypeptide to the inner wall of the import channel and subsequently by Tim40/Mia40 binding in the IMS at the outlet of the channel (Fig. 5, step 2). Further translocation of the substrate polypeptide could be partly driven by the increase in the conformational entropy in the IMS by the mechanism called entropic pulling^49^. Presence of a long disordered segment (the DUF domain for Mic19) in front of the TIM40/MIA substrate domain (the CHCH domain for Mic19) would further increase the conformational entropy of Mic19 in the cytosol, which should make the entry of the CHCH domain into the Tom40 import channel much less favorable (Fig. 5, step 3). However, binding of the N-terminal segment to Tom20 as well as the OM through a myristoyl group will make the DUF domain closely apposed to the membrane, thereby increasing the excluded-volume constraint between the DUF domain and the membrane (Fig. 5, step 4). This will counteract the increase in the conformational entropy arising from the attached DUF domain of Mic19 in the cytosol, thereby driving the entry of the CHCH domain of Mic19 into the Tom40 channel (Fig. 5, step 5). After this “entropic pushing”, the CHCH domain will be trapped by Tim40 in the IMS, which should function as the *trans* side trap, like canonical substrates for the TIM40/MIA pathway substrates (Fig. 5, step 6). Dissociation of the myristoyl group from Tom20 in the cytosol followed by subsequent binding to the TOB/SAM complex in the IMS^23^ may also contribute to driving the further translocation of Mic19 across the OM. Efficient sequestration of the unfolded DUF domain from the cytosolic side of the outer membrane may be also important for preventing it from activation of the mitochondrial stress response due to accumulation of unfolded proteins on the mitochondrial surface^50^. Indeed, replacement of the motif for myristoylation in Mic19 with the one for more hydrophobic palmitoylation increased binding to mitochondria, but decreased import efficiency (data not shown), suggesting that optimized hydrophobicity of the N-terminally attached acyl chain is important for efficient binding and dissociation of Mic19 from the mitochondrial surface. The scenario shown in Fig. 5 can be further tested experimentally, and the enigmatic function of the DUF domain should be addressed in future studies. The DUF domain could facilitate optimized distribution of Mic19 to distinct regions of the IM, in addition to crista junction, for interactions with outer membrane components like Tob55/Sam50 or with inner membrane components like CoxIV after reaching the intermembrane space^22^.

## Conclusion

As an essential step toward understanding of the mechanisms of assembly of the MICOS complex and the following crista junction formation, we analyzed here the import pathways of the six yeast MICOS subunits into mitochondria. Among those MICOS subunits, only Mic60 possesses a cleavable presequence followed by a TM segment. Mic60 was found to follow the presequence pathway via the TOM40 complex and TIM23 complex, which exclusively requires ΔΨ, to be inserted laterally into the IM by the stop-transfer mechanism. Mic10, Mic12, Mic26, and Mic27 also follow the TIM23 pathway for import, but depending only mildly on ΔΨ. In contrast to these 5 MICOS subunits, Mic19 consists of an N-terminal myristoylation domain followed by a DUF domain and a CHCH domain, and its import did not depend on the TIM23 complex or ΔΨ, but instead used the TIM40/MIA pathway for the import of the CHCH domain into the IMS. The N-terminal attachment of the DUF domain or an unrelated long unfolded segment to a TIM40/MIA pathway substrate reduced its import competence, but that the presence of the myristoylation domain in front of the unfolded segment circumvented the import-impairing effect (Fig. 4). Although the presence of a long unfolded segment (the DUF domain for Mic19) in front of the TIM40/MIA substrate domain (the CHCH domain for Mic19) would increase the conformational entropy of Mic19 in the cytosol, which is unfavorable for the entry of the CHCH domain into the Tom40 import channel, this entropy decrease will be counteracted by binding of the myristoyl group to Tom20 and the OM, which would increase the excluded-volume constraint between the DUF domain and the TOM complex and the OM (Fig. 5). We thus propose this “entropy pushing” as a previously overlooked, new mechanism of importing proteins with a long disordered segment into mitochondria. Since proteins can be often myristoylated at their N-terminus, this mechanism could operate for other proteins destined for other organelles as well as mitochondria.

## Materials and Methods

### Yeast strains

Yeast strains used in this study are listed in (Table S1A). The *MIC19* gene was deleted with kanMX4, which was amplified by PCR with pBluescript-kanMX4 as a template. A yeast strain expressing OsTIR1 was made by integration of pNHK53 linearized with StuI into the *URA3* locus. PCR cassettes for the AID*-9xMYC tag or 3xmini-AID tag were amplified from pKan-AID*-9xMYC or pMK151, respectively, and were integrated into the 3′-end of the *TOM20* locus of the OsTIR1 strain. DNA construction for attaching the 3 × FLAG-tag to the C-terminus of Tim40 was performed by homologous recombination of the W303-1A strain using appropriate gene cassettes from pFA6a-FLAG-kanMX6.

### Plasmids

Plasmids and PCR primers used in this study are listed in Tables S1B,C, respectively. Plasmids for *in vitro* translation were constructed by standard molecular cloning techniques with materials listed in Table S1. Site-directed mutagenesis and partial deletion mutagenesis were performed by QuickChange.

### Growth media

Yeast cells were grown in lactate medium (0.3% yeast extract, 0.05% glucose, 0.05% CaCl_2_, 0.05% NaCl, 0.06% MgCl_2_, 0.1% KH_2_PO_4_, 0.1% NH_4_Cl and 2% lactic acid, pH 5.6), YPD (1% yeast extract, 2% polypeptone, and 2% glucose), YPGly (1% yeast extract, 2% polypeptone, 3% glycerol, and 0.05% glucose), YPLac (1% yeast extract, 2% polypeptone, and 3% lactate pH 5.6), or SCLac (0.67% (w/v) yeast nitrogen base without amino acids, 0.5% (w/v) vitamin assay casamino acid, and 2% lactic acid, pH 5.6).

### *In vitro* protein import into isolated mitochondria

Radiolabeled proteins were synthesized in a cell-free transcription/translation system with rabbit reticulocyte lysate in the presence of [^35^S]-methionine. Import of radiolabeled precursor proteins into isolated yeast mitochondria was performed at 25 or 30 °C in import buffer (250 mM sucrose, 10 mM MOPS-KOH, pH 7.4, 80 mM KCl, 2 mM ATP, 20 mM NADH, 12 mM creatine phosphate, 120 μg/ml creatine kinase, 2 mM methionine, 5 mM MgCl_2_, 2 mM DTT, 2.5 mM KPi, and 1% BSA). Import reactions were stopped by addition of 500 μl ice-cold SEM buffer (250 mM sucrose, 10 mM MOPS-KOH, pH 7.2, 1 mM EDTA). To dissipate ΔΨ, mitochondria were pre-incubated in import buffer with 10 μg/ml valinomycin, and import was performed in the presence of valinomycin.

### Analysis of the mixed disulfide intermediate with Tim40-FLAG

Radiolabeled Mic19C146S was imported into mitochondria with Tim40-FLAG for 20 min at 25 °C. The import reactions were stopped by adding 500 μl of ice-cold SEM buffer containing 50 mM 2-iodoacetamide (IAA). The mitochondria were re-isolated by centrifugation at 20,000 × *g* for 10 min at 4 °C and solubilized with 1% digitonin in 200 μl of solubilization buffer (20 mM Tris-HCl, pH 7.4, 150 mM NaCl, and 1 mM phenylmethylsulfonyl fluoride (PMSF)) with 50 mM IAA for 30 min on ice. After clarifying spin at 20,000 × *g* for 5 min at 4 °C, the supernatant was mixed with 800 μl of solubilization buffer with 50 mM IAA and 10 μl of anti-FLAG M2-agarose and rotated gently for 2 hours at 4 °C. The agarose resin was washed three times with 1 ml wash buffer (0.2% digitonin, 20 mM Tris-HCl, pH 7.4, 150 mM NaCl, 50 mM IAA, and 1 mM PMSF). Bound proteins were eluted with SDS-sample buffer containing 5 M urea and analyzed by SDS-PAGE in the presence of 5 M urea, but without 5% β-mercaptoethanol.

### *In vitro* myristoylation

Radiolabeled proteins (Mic19(WT)-FLAG, Mic19(G2A)-FLAG or Mic19(Δ20)-FLAG) were synthesized in a cell-free transcription/translation system with rabbit reticulocyte lysate in the presence of [^3^H]-myristic acid or [^35^S]-methionine. The translation products (50 μl) were mixed with 950 μl of lysis buffer (20 mM Tris-HCl, pH7.4, 150 mM NaCl, 0.5% TritonX-100, 0.5 mM EDTA and 1 mM PMSF) containing 20 μl anti-FLAG M2-agarose and incubated for 2 hours at 4 °C. The agarose resin was washed three times with 1 ml lysis buffer, and bound proteins were eluted by boiling in SDS-sample buffer and analyzed by SDS-PAGE (Fig. 3A).

### Tom20 binding assay

The cytosolic receptor domain of rat Tom20 (Tom20^sol^-His) was prepared as reported previously^45,51^ with minor modifications. The *E. coli* strain BL21(DE3) transformed with the pET-22b plasmid (Merck Millipore) containing the gene for Tom20^sol^-His, the cytosolic receptor domain of Tom20 from *Rattus norvegicus* (residues 51–145) with a hexa-histidine tag at the C-terminus, was cultured in LB medium containing 50 μg/ml ampicillin at 37 °C until OD_600_ reached 0.5. Protein expression was induced by 0.5 mM isopropyl β-D-1-thiogalactopyranoside for 16 hours at 16 °C. Then cells were collected and re-suspended in 20 mM Tris-HCl, pH7.4, containing 300 mM NaCl followed by cell disruption by sonication. The cell lysate was centrifuged at 35,000 × *g* for 40 min at 4 °C to remove cell debris and unbroken cells, and the resulting supernatant was subjected to Ni-NTA chromatography (QIAGEN). Tom20^sol^-His was eluted with 500 mM imidazole and further purified by gel-filtration chromatography on a HiLoad 26/600 Superdex 200 pg column (GE-Healthcare) with 20 mM Tris-HCl, pH7.4, containing 150 mM NaCl. Fractions containing Tom20^sol^-His were pooled and the protein was concentrated with Amicon-Ultra 15 (3 K cutoff, Merck Millipore).

To prepare immobilized Tom20^sol^-His, 10 μl of Ni-NTA resin (QIAGEN) was incubated with 0.0 or 0.5 µg of purified Tom20^sol^-His for 1 hour at 4 °C. One mg protein of mitochondria were solubilized with 1% digitonin in 200 μl of solubilization buffer (20 mM Tris-HCl, pH 7.4, 300 mM NaCl, 10% (w/v) glycerol, and 1 mM PMSF) for 30 min on ice. After clarifying spin at 20,000 × *g* for 5 min at 4 °C, the supernatant was mixed with 10 µl of immobilized Tom20^sol^-His and rotated gently for 14 hours at 4 °C. Then the agarose Ni-NTA resin was washed three times with 1 ml wash buffer (1% digitonin, 20 mM Tris-HCl, pH 7.4, 50 mM NaCl, 10% (w/v) glycerol, 1 mM PMSF and 50 mM imidazole), and bound proteins were eluted with 30 μL of elution buffer (0.2% Triton X-100, 20 mM Tris-HCl, pH 7.4, 50 mM NaCl, 10% (w/v) glycerol, 1 mM PMSF and 500 mM imidazole) and analyzed by SDS-PAGE (Fig. 4F).

### Cell fractionation

Yeast cells were grown in SCLac-Trp at 30 °C for 14 h until the OD_600_ reaches 0.8. 0.1 g of the cells were incubated in alkaline buffer (100 mM Tris-SO_4_, pH 9.6, 10 mM DTT) at 30 °C for 20 min. Then, cells were collected and suspended in 1.2 M sorbitol buffer (1.2 M sorbitol, 20 mM Tris-HCl, pH 7.4) with 2 units/ml Zymolyase 20 T for incubation for 45 min at 30 °C. The resulting spheroplasts were collected, suspended in 200 µl of 0.7 M sorbitol buffer (20 mM Tris-HCl, pH 7.4, 5 mM EDTA, 1 mM PMSF, protease inhibitor cocktail) containing 200 µl of glass beads, and vortexed twice for 1 min with an interval of 1 min on ice. Unbroken cells were removed by centrifugation at 1,000 × *g* for 5 min at 4 °C, and 300 µl of the supernatant was collected. 150 µl of the supernatant was used as total lysate. Remaining150 µl of the supernatant was centrifuged at 20,000 × *g* for 10 min at 4 °C and the resulting supernatant (100 μl) and pellet were used as membrane and cytosol fractions, respectively. The pellet was further washed three times with SEM buffer. Total lysate and membrane and cytosol fractions were solubilized in 225, 225, and 150 µl, respectively, of SDS sample buffer and proteins in the same relative portions for each fraction were analyzed by SDS-PAGE (Fig. 3B).

### Equipment and settings

Radiolabeled proteins were analyzed by SDS-PAGE followed by radioimaging with Imaging Plate and image analyzers, Typhoon FLA-7000 (GE Healthcare), Typhoon FLA-9500 (GE Healthcare), and Typhoon 9200 (GE Healthcare). Signals of immunoblotting were detected with image analyzers, Typhoon FLA-7000 (GE Healthcare), Typhoon 9200 (GE Healthcare), Storm 860 (Molecular Dynamics), and Amersham Typhoon 5 Biomolecular imager (GE Healthcare). Blots/gel images were analyzed by using software, ImageQuant TL (GE Healthcare), Adobe Photoshop, Adobe Illustrator, multi gauge (Fuji Film), and Image J (Wayne Rasband).

### Miscellaneous

For treatment of mitochondria with proteinase K (PK), mitochondria were incubated with 50 μg/ml PK for 20 min on ice, and the reactions were stopped by addition of 1 mM PMSF. After reisolation, mitochondria were washed with SEM buffer, and proteins were analyzed by SDS-PAGE.

## Supplementary information

Supplementary Figures and Table
