# Supplementary material for: Myristoyl group-aided protein import into the mitochondrial intermembrane space
Source: Sci Rep. 2019 Feb 4;9:1185. doi: 10.1038/s41598-018-38016-1 (PMC6362269; doi:10.1038/s41598-018-38016-1)
Supplement: Supplementary file 1 — Supplementary Figures and Table [file 41598_2018_38016_MOESM1_ESM.pdf]

(Revised version)

Supplementary Information

Myristoyl group-aided protein import into the mitochondrial intermembrane space

Eri Ueda, Yasushi Tamura, Haruka Sakaue, Shin Kawano, Chika Kakuta, Shunsuke Matsumoto, and Toshiya Endo

Supplementary Figures S1-S9

Supplementary Table S1

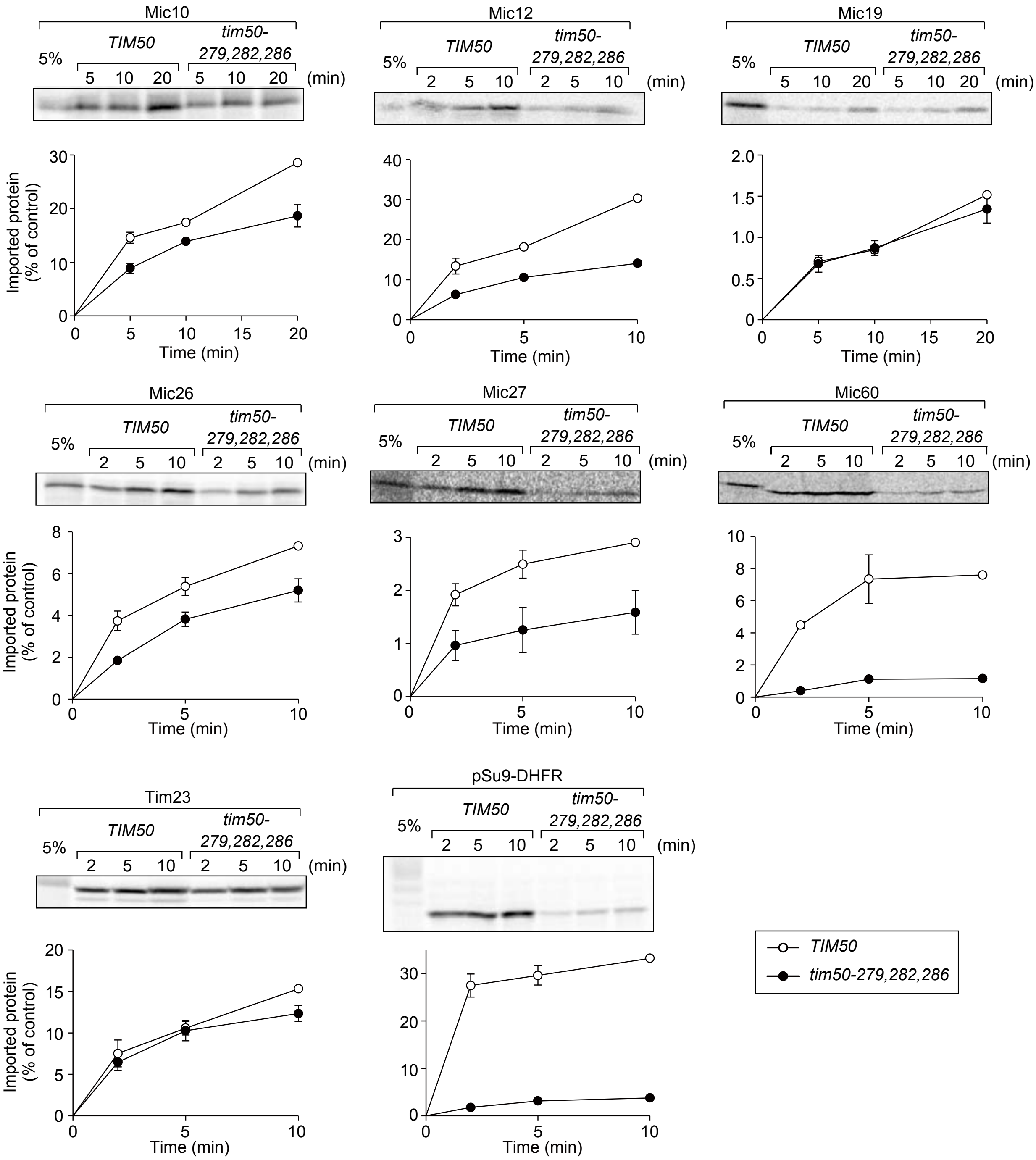

Figure S1. Import of MICOS subunits except for Mic19 depends on Tim50 of the TIM23 complex. The indicated radiolabeled proteins were incubated with *TIM50* mitochondria (open circles) or *tim50-279,282,286* mitochondria (closed circles; Tamura et al., 2009) for the indicated times at 25°C. Imported proteins were analyzed as in Fig. 2 E. Full-length gel images are presented in Supplementary Figure S8.

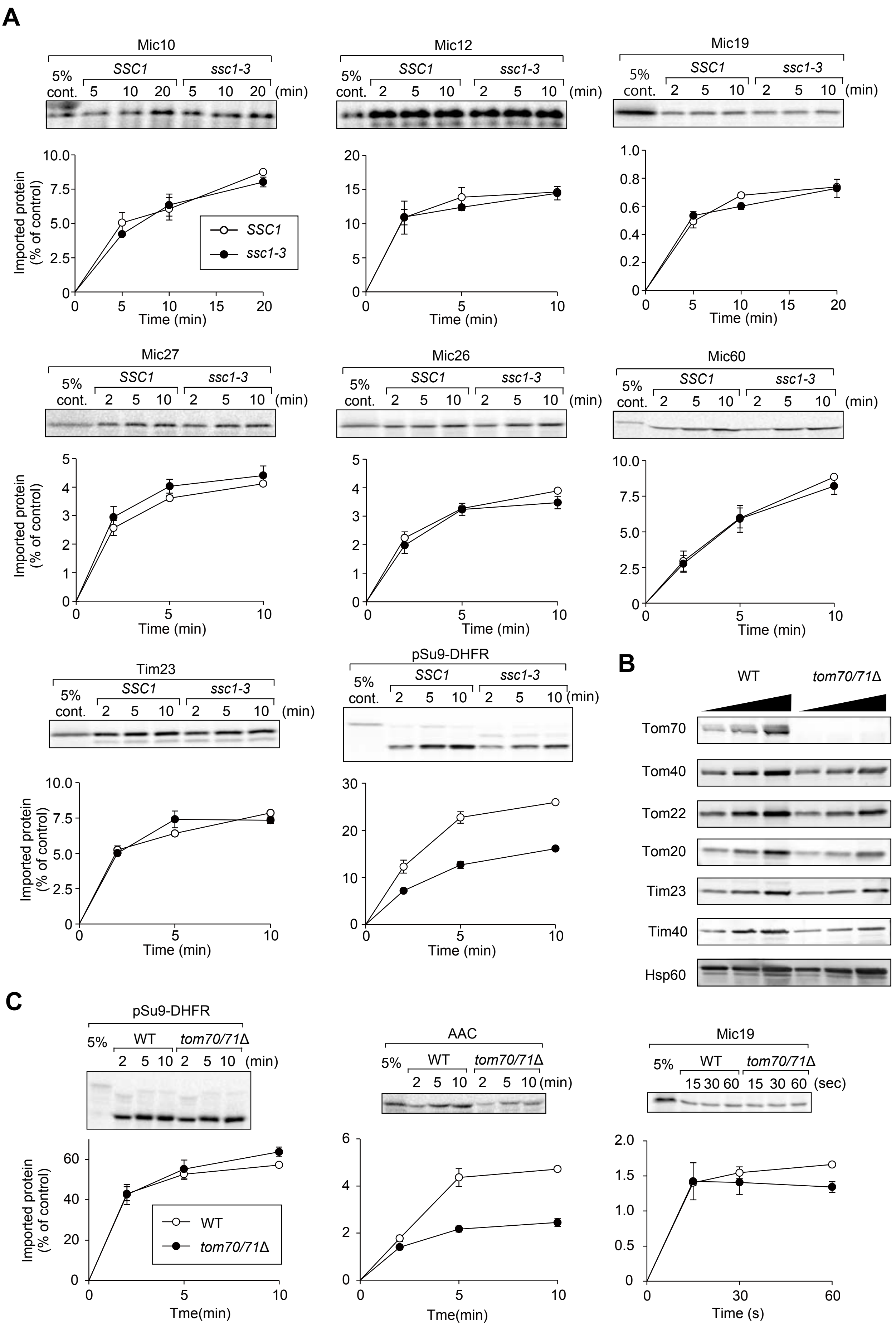
A
Mic10
5% cont.
SSC1
ssc1-3
5 10 20 5 10 20 (min)
Imported protein (% of control)
Time (min)
SSC1
ssc1-3
Mic12
2 5 10 2 5 10 (min)
Mic19
Mic27
Mic26
Mic60
Tim23
pSu9-DHFR
B
WT
tom70/71Δ
Tom70
Tom40
Tom22
Tom20
Tim23
Tim40
Hsp60
C
pSu9-DHFR
5%
WT
tom70/71Δ
2 5 10 2 5 10 (min)
Tme(min)
AAC
Mic19
15 30 60 15 30 60 (sec)
Time (s)

Figure S2.

Figure S2 (continued). Import of MICOS subunits or Mic19 does not require Ssc1 or Tom70/71, respectively. (A) The indicated radiolabeled proteins were incubated with *SSC1* mitochondria (open circles) or *ssc1-3* mitochondria (closed circles; Gambill et al., 1993) for the indicated times at 30°C. Imported proteins were analyzed as in Fig. 2 E. (B) The indicated proteins were analyzed by SDS-PAGE followed by immunoblotting for wild-type mitochondria (WT) and those lacking Tom70 and Tom71 (*tom70/71*Δ), which were isolated after cultivation in lactate medium (+0.05% glucose) at 30°C. (C) The indicated radiolabeled proteins were incubated with WT (open circles) or *tom70/71*Δ (closed circles) mitochondria (0.5 mg of protein/ml) at 25°C for the indicated times. Imported proteins were analyzed as in Fig. 2E. Full-length gel images are presented in Supplementary Figure S9.

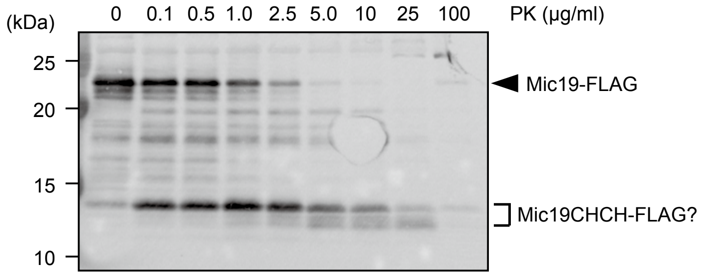

Mic19-FLAG	22 kDa
CHCH-FLAG	9.8 kDa

Figure S3. Mitochondria containing Mic19 with C-terminal FLAG tag (Mic19-FLAG) were solubilized with1% Triton X100 and digested with different concentrations of proteinase K (PK) for 20 min on ice. The reaction was stopped by addition of PMSF. Proteins were TCA precipitated and subjected to SDS-PAGE and immunoblotting with the anti-FLAG antibody. Calculated molecular weigts of Mic19-FLAG and the CHCH domain-FLAG were 22 kDa and 9.8 kDa, respectively.

1B

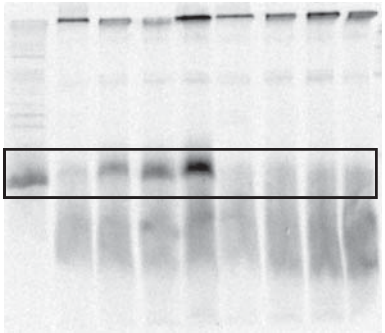

$[^{35}S]$Mic10

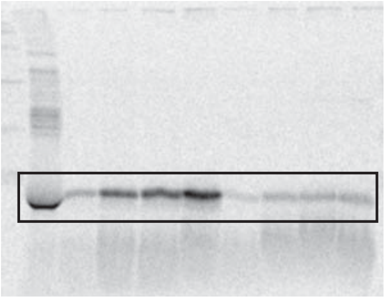

$[^{35}S]$Mic12

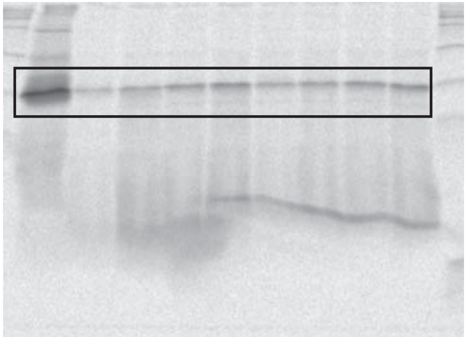

$[^{35}S]$Mic19

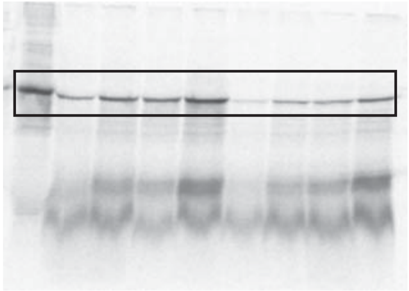

$[^{35}S]$Mic26

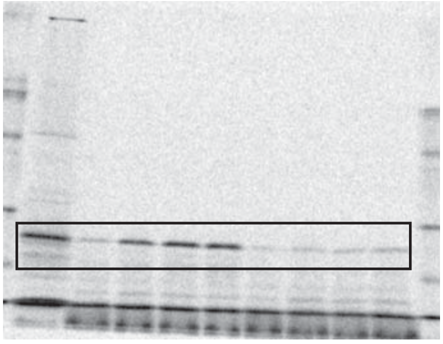

$[^{35}S]$Mic27

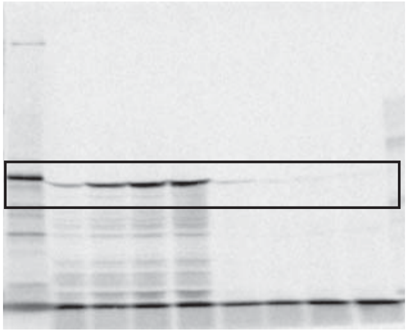

$[^{35}S]$Mic60

Figure S4. Full-length gel images for Fig. 1B

2A

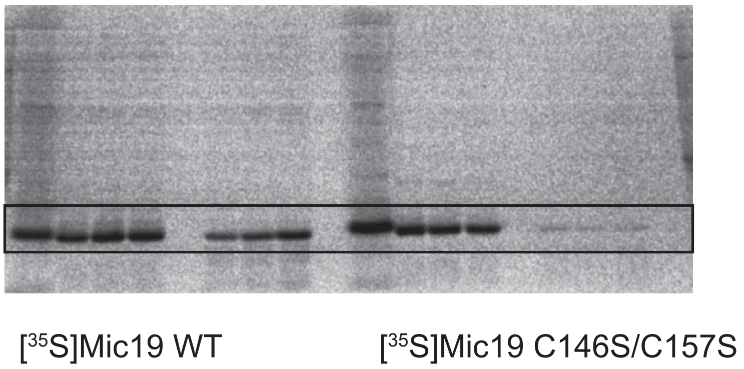

2D

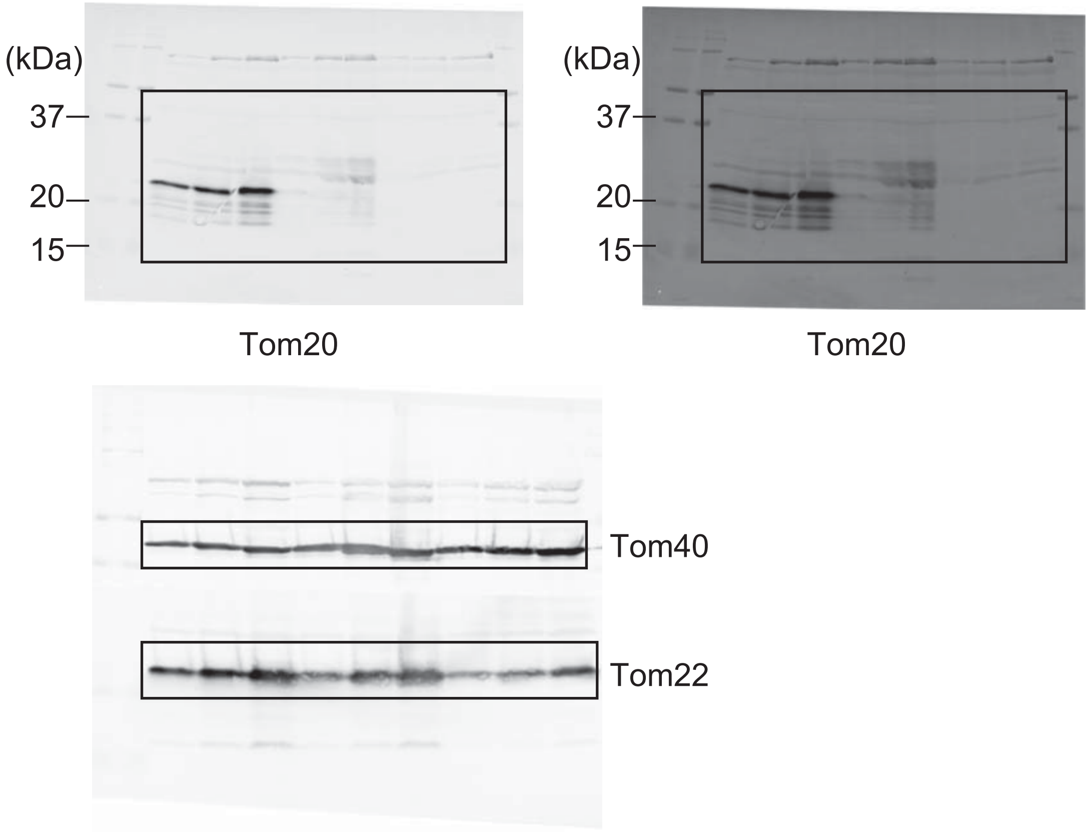

2E

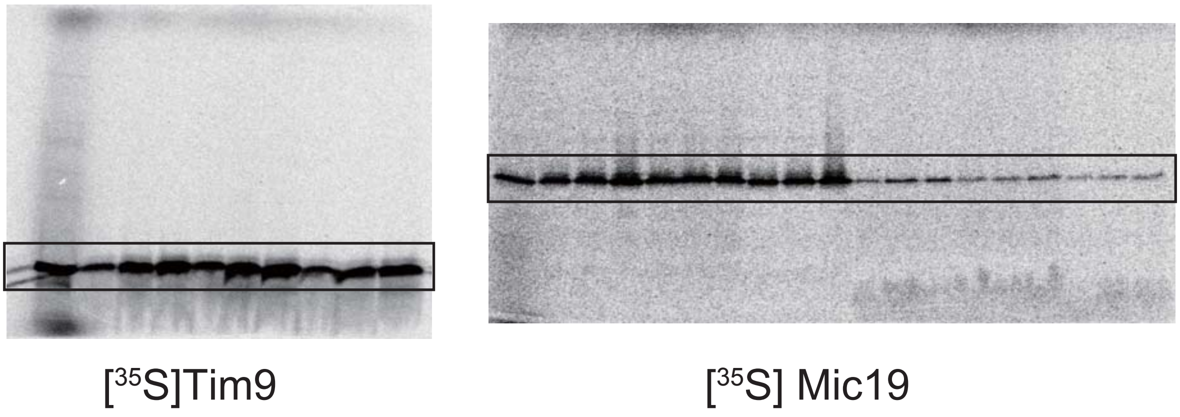

Figure S5. Full-length gel images for Fig. 2A, 2D, and 2E.

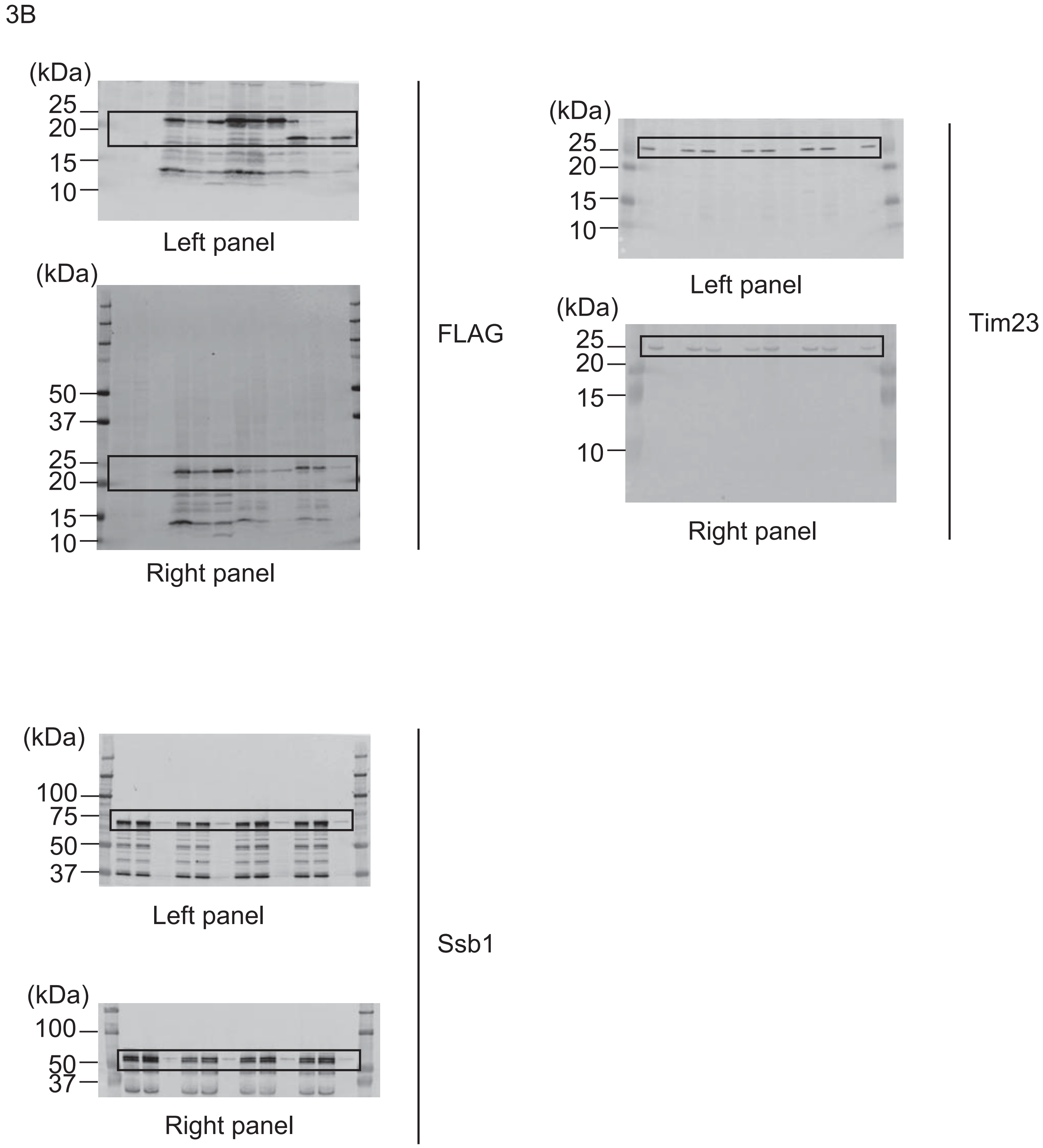

Figure S6. Full-length gel images for Fig. 3B

3C

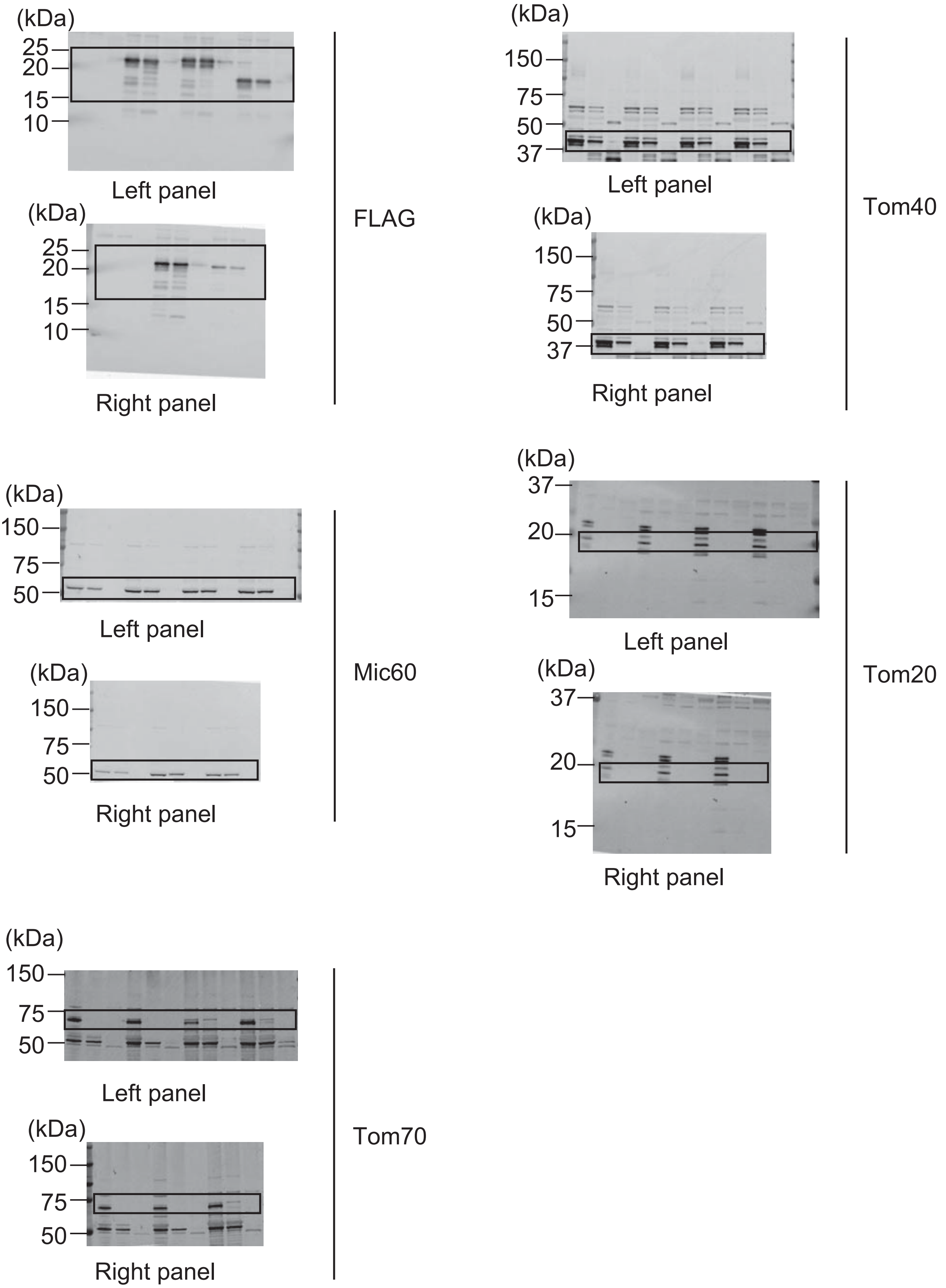

Figure S6 (continued). Full-length gel images for Fig. 3C

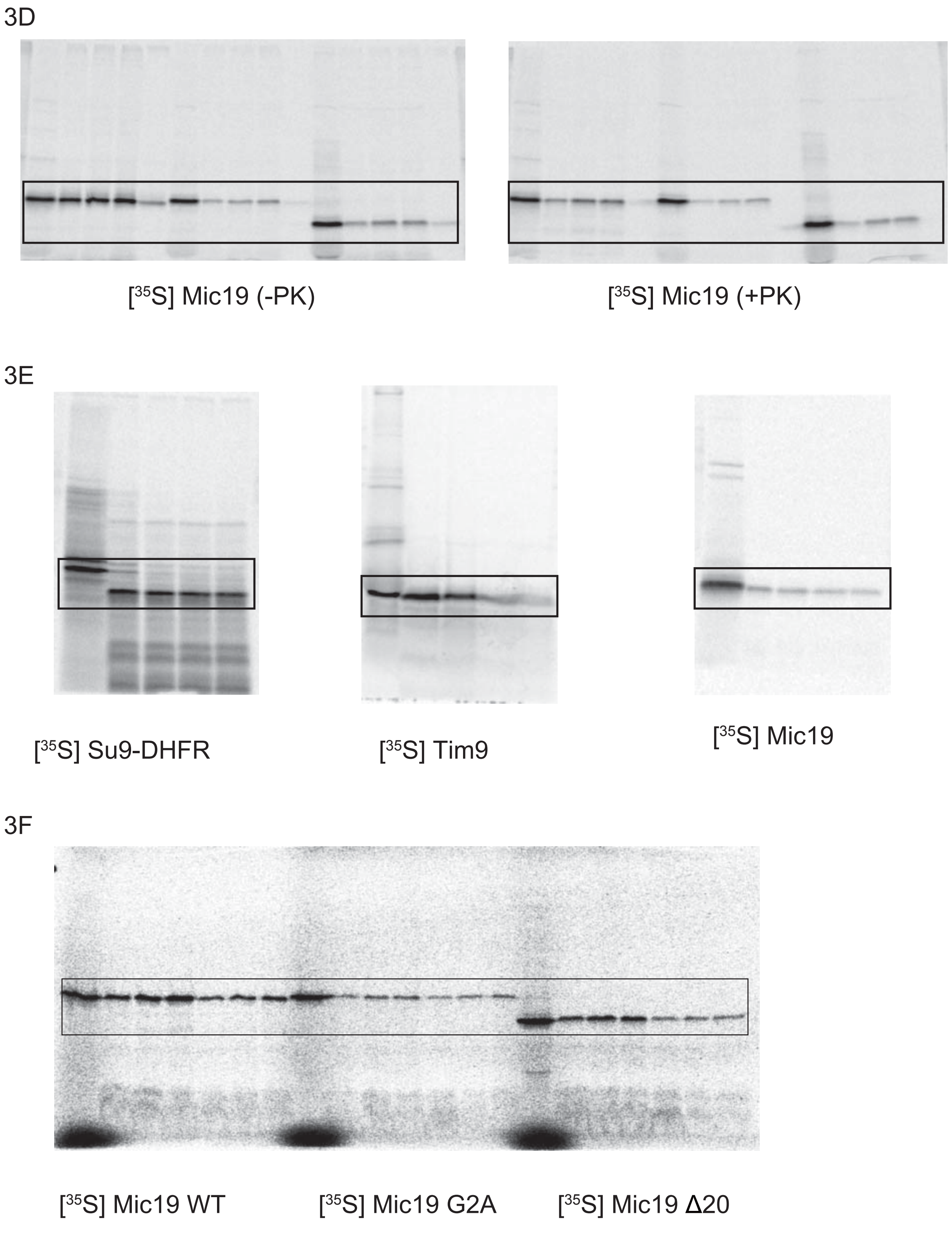

Figure S6 (continued). Full-length gel images for Fig. 3D, 3E, and 3F

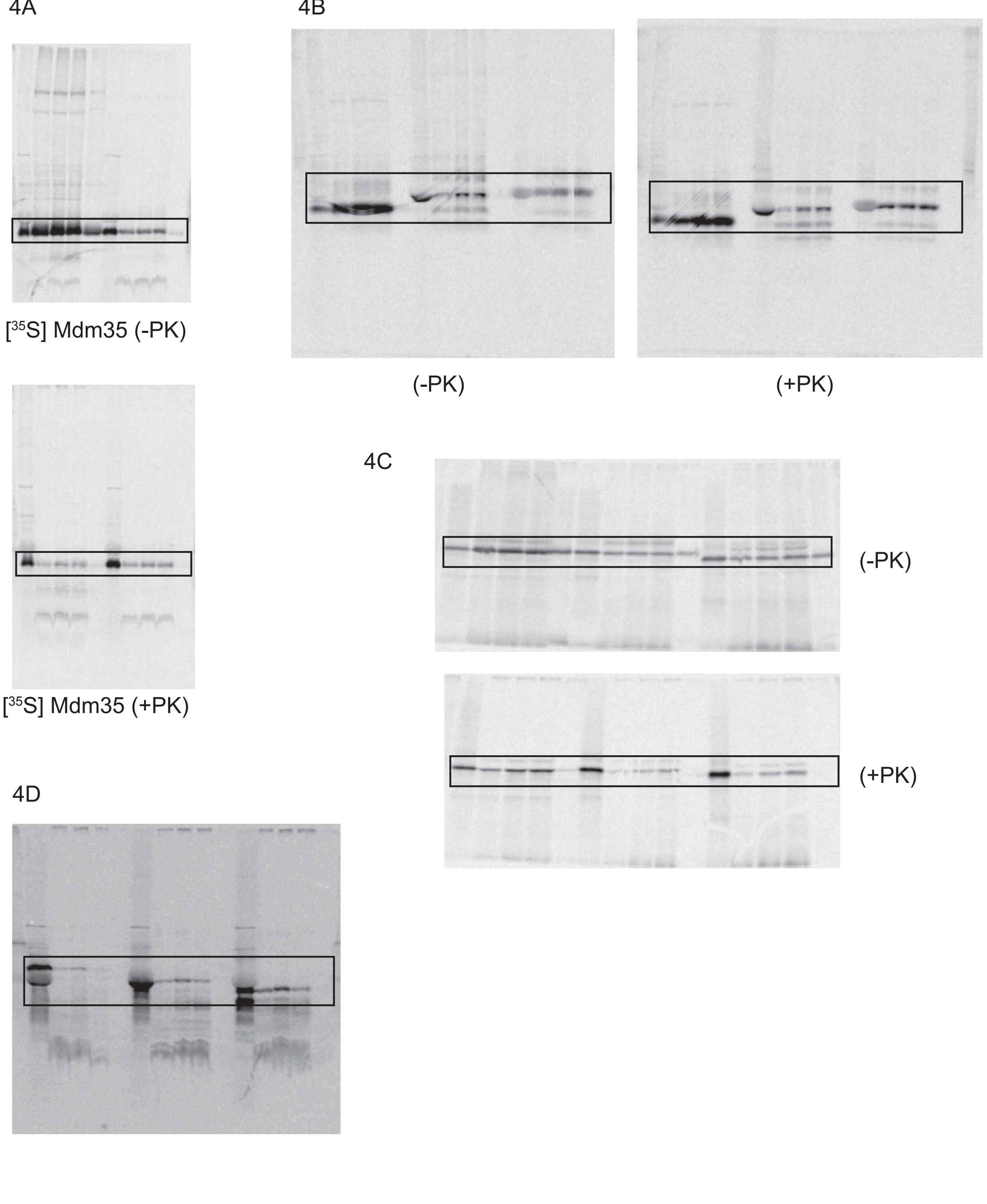

Figure S7. Full-length gel images for Fig. 4A, 4B, and 4C.

4E

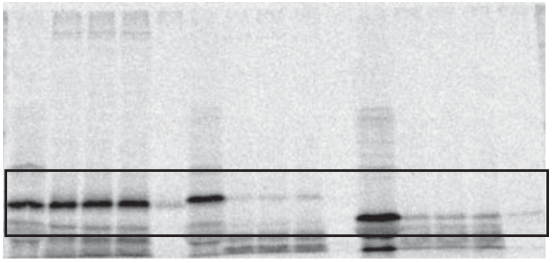

(-PK)

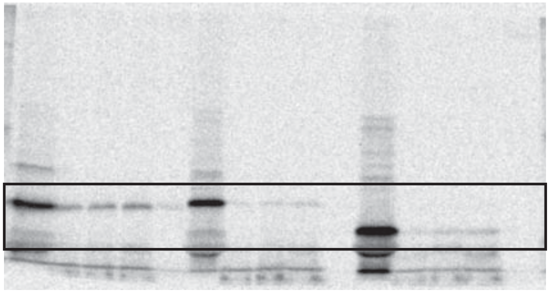

(+PK)

4F

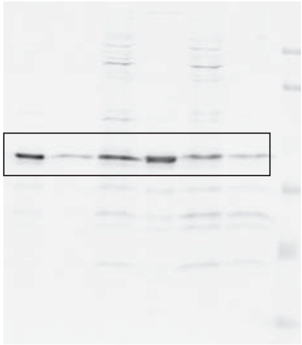

FLAG

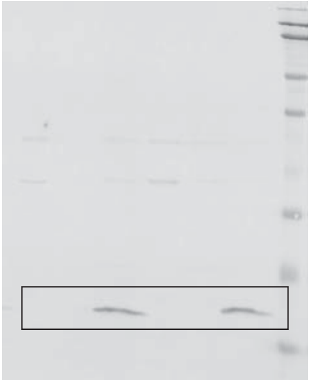

His

Figure S7 (continued). Full-length gel images for Fig. 4E and 4F.

S1

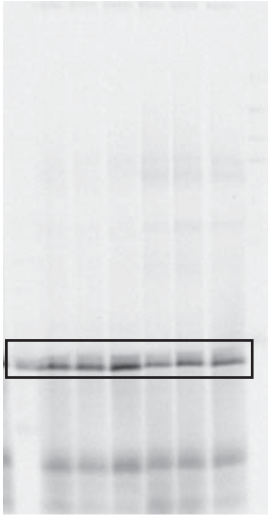

[^{35}S]Mic10

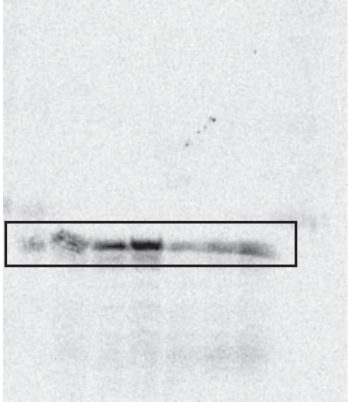

[^{35}S]Mic12

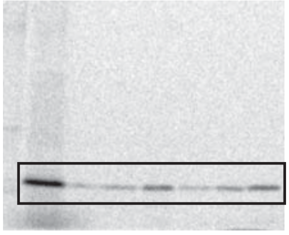

[^{35}S]Mic19

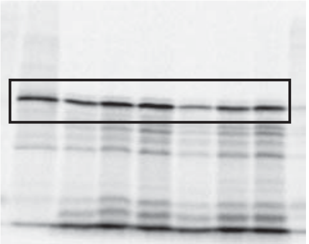

[^{35}S]Mic26

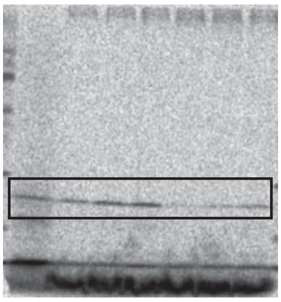

[^{35}S]Mic27

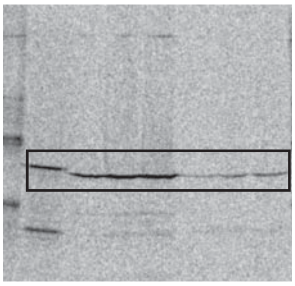

[^{35}S]Mic60

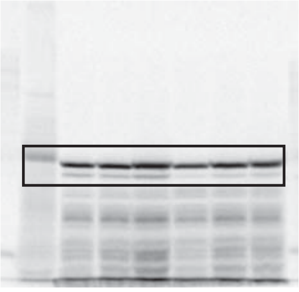

[^{35}S]Tim23

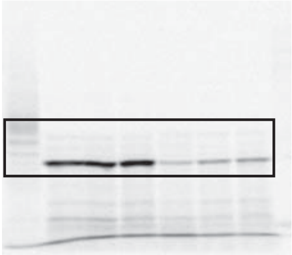

[^{35}S]Su9-DHFR

Figure S8. Full-length gel images for Fig.S1.

S2A

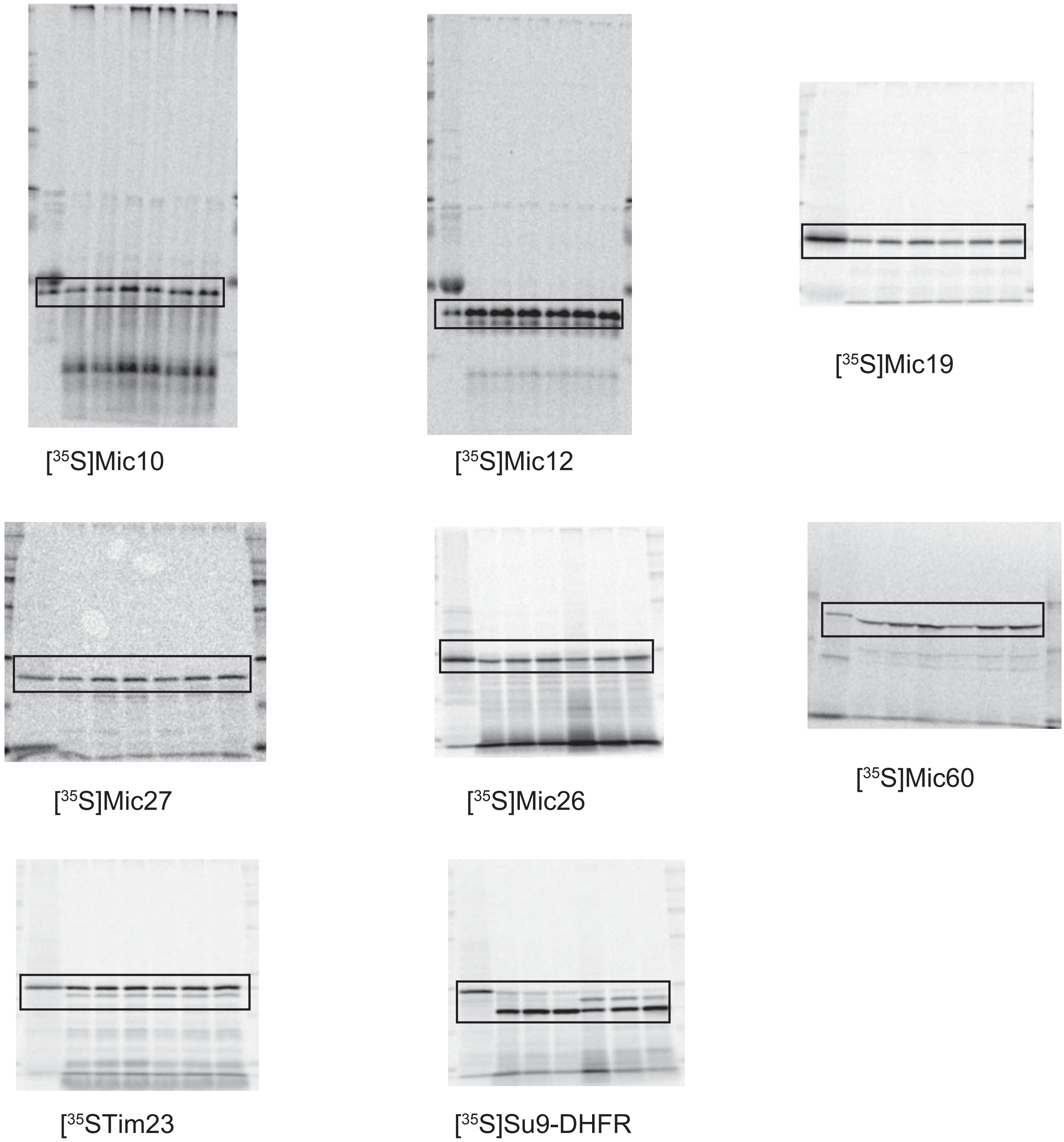

Figure S9. Full-length gel images for Fig. S2A.

S2B

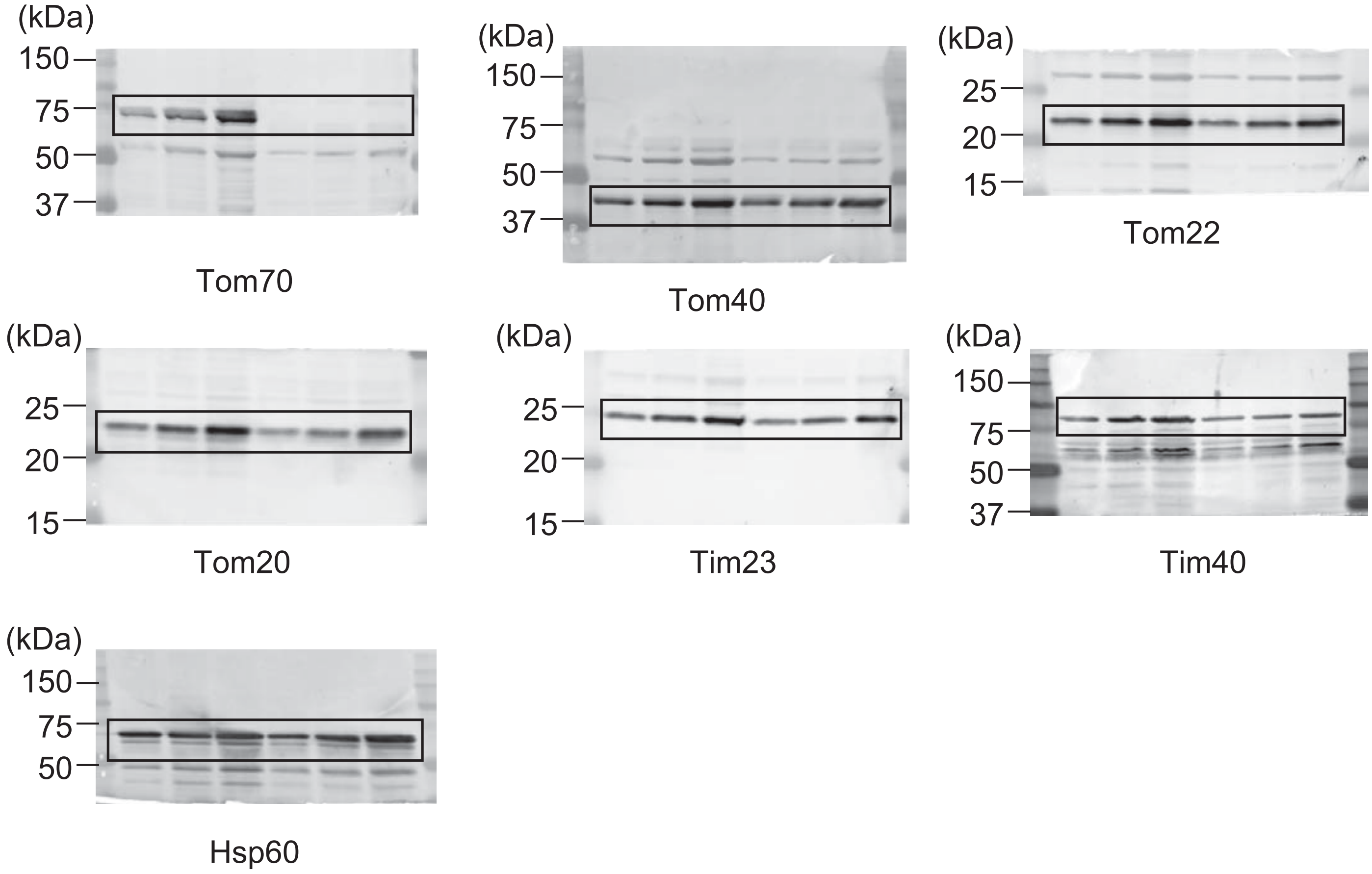

S2C

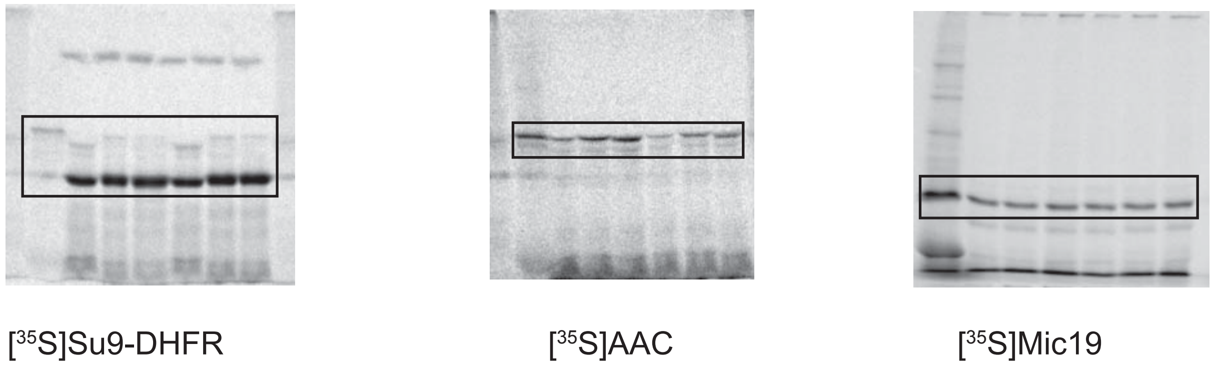

Figure S9 (continued). Full-length gel images for Fig. S2B and S2C.

Table S1A. Strain list

Strain	Genotype	Source
W303-1A	*MATa ade2-1 his3-11, 15 ura3-1 leu2-3, 112 trp1-1 can1-100*	
D273-10B	*MATa*	
TIM50	*MATa ade2 his3 ura3 leu2 trp1 can1 tim50Δ::CgHIS3 [pRS314-Tim50-WT]*	Tamura *et al.* , 2009
tim50-278,282,286	*MATa ade2 his3 ura3 leu2 trp1 can1 tim50Δ::CgHIS3 [pRS314-Tim50-279,282,286]*	Tamura *et al* ., 2009
SSC1	*MATa his4-713 lys2 ura3-52 Δtrp1 leu2-3, 112*	Gambil *et al.* , 1993
ssc1-3	*MATa his4-713 lys2 ura3-52 Δtrp1 leu2-3, 112 ssc1-3 ::CgLEU2*	Gambil *et al.* , 1993
tom70/71 Δ	*MATa ade2-1 leu2-3 his3-11,15 trp1-1 ura3-1 can1-100 tom70Δ::TRP1 tom71Δ::HIS3*	Kondo-Okamoto *et al.* , 2008
Mic19 Δ	*MATa ade2-1 his3-11, 15 ura3-1 leu2-3, 112 trp1-1 can1-100 Mic19Δ ::kanMX*	This study
Mic19 ΔMic19(xx)-FLAG	*MATa ade2-1 his3-11, 15 ura3-1 leu2-3, 112 trp1-1 can1-100 Mic19Δ ::kanMX [pRS314-Mic19(xx)-FLAG]*	This study
OsTIR1	*MATa ade2-1 his3-11, 15 ura3-1 leu2-3, 112 trp1-1 can1-100 ura3::ADH1p-OsTIR1-9xMYC*	This study
tom20-AID-9xMYC*	*MATa ade2-1 his3-11, 15 ura3-1 leu2-3, 112 trp1-1 can1-100 ura3::ADH1p-OsTIR1-9xMYC tom20-AID*-9xMYC-KanM*	This study
tom20-3xmini-AID	*MATa ade2-1 his3-11, 15 ura3-1 leu2-3, 112 trp1-1 can1-100 ura3::ADH1p-OsTIR1-9xMYC tom20-3xmini AID-KanMX*	This study

*XX:Mic19 variants

Table S1B. Plasmid list

Plasmid name	Expressed protein	Promoter	Vector	primer	Site	Template DNA or Source
pGEM4Z-Mic10	Mic10	SP6	pGEM4Z	EcoRI-Mic10(-50)-F<br>BamHI-Mic10-stop-R	EcoRI/BamHI	yeast genomic DNA
pGEM4Z-Mic12	Mic12	SP6	pGEM4Z	EcoRI-Mic12(-50)-F<br>BamHI-Mic12-stop-R	EcoRI/BamHI	yeast genomic DNA
pGEM4Z-Mic19	Mic19	SP6	pGEM4Z	SacI-Mic19(-50)-F<br>BamHI-Mic19-stop-R	SacI/BamHI	yeast genomic DNA
pTNT-Mic27	Mic27	SP6	pTNT	EcoRI-Mic27(-10)-F<br>SalI-Mic27-stop-R	EcoRI/BamHI	yeast genomic DNA
pGEM4Z-Mic26	Mic26	SP6	pGEM4Z	EcoRI-Mic26(-50)-F<br>BamHI-Mic26-stop-R	EcoRI/BamHI	yeast genomic DNA
pTNT-Mic60	Mic60	SP6	pTNT	EcoRI-Mic60-F<br>SalI-Mic60-stop-R	EcoRI/BamHI	yeast genomic DNA
pGEM4Z-Mic19C146S	Mic19 C146S	SP6	pGEM4Z	Mic19-C146S-F<br>Mic19-C146S-R	N/A	pGEM4Z-Mic19
pGEM4Z-Mic19C157S	Mic19 C157S	SP6	pGEM4Z	Mic19-C157S-F<br>Mic19-C157S-R	N/A	pGEM4Z-Mic19
pGEM4Z-Mic19C146S/C157S	Mic19 C146S/C157S	SP6	pGEM4Z	Mic19-C146S-F<br>Mic19-C157S-R	N/A	pGEM4Z-Mic19
pGEM4Z-Mic19G2A	Mic19 G2A	SP6	pGEM4Z	Mic19-G2A-F<br>Mic19-G2A-R	N/A	pGEM4Z-Mic19
pGEM4Z-Mic19Δ20	Mic19 Δ20	SP6	pGEM4Z	Mic19(Δ20)-F<br>SP6-R	N/A	pGEM4Z-Mic19
pGEM4Z-Mic19G2A/C146S/C157S	Mic19 G2A/C146S/C157S	SP6	pGEM4Z	Mic19-G2A-F<br>Mic19-G2A-R	N/A	pGEM4Z-Mic19C146S/C157S
pTNT-Tim9	Tim9	SP6	pTNT	pTNT-EcoRI-Tim9-F<br>pTNT-SalI-Tim9-R	EcoRI/SalI	yeast genomic DNA
pTNT-Mic19wt(1-20)-Tim9	Mic19(1-20)-Tim9	SP6	pTNT-Tim9	EcoRI-Mic19WT(1-)-F<br>EcoRI-Mic19(-20)-R	EcoRI	pGEM4Z-Mic19
pTNT-Mic19G2A(1-20)-Tim9	Mic19 G2A(1-20)-Tim9	SP6	pTNT-Tim9	EcoRI-Mic19G2A(1-)-F<br>EcoRI-Mic19(-20)-R	EcoRI	pGEM4Z-Mic19
pTNT-Mic19WT(1-127)-Tim9	Mic19(1-127)-Tim9	SP6	pTNT-Tim9	EcoRI-Mic19WT(1-)-F<br>EcoRI-Mic19(-127)-R	EcoRI	pGEM4Z-Mic19
pTNT-Mic19G2A(1-127)-Tim9	Mic19 G2A(1-127)-Tim9	SP6	pTNT-Tim9	EcoRI-Mic19G2A(1-)-F<br>EcoRI-Mic19(-127)-R	EcoRI	pGEM4Z-Mic19G2A
pTNT-Mic19(21-127)-Tim9	Mic19 (21-127)-Tim9	SP6	pTNT-Tim9	EcoRI-Mic19(21-) -F<br>EcoRI-Mic19(-127)-R	EcoRI	pGEM4Z-Mic19Δ20
pTNT-Mdm35	Mdm35	SP6	pTNT	EcoRI-Mdm35-F<br>SalI-Mdm35-R	EcoRI/SalI	yeast genomic DNA
pTNT-Mdm35G2A	Mdm35 G2A	SP6	pTNT	Mdm35G2A-F<br>SP6-R	N/A	pTNT-Mdm35
pGEM4Z-Mic19WT(1-20)-XhoI-(21-127)-KpnI-(128-170	Mic19(1-20)-LE-Mic19(21-127)-GT-Mic19(128-170)	SP6	pGEM4Z	Mic19(20)-XhoI-Mic19(21)-F<br>Mic19(127)-kpnI-Mic19(128)-R	N/A	pGEM4Z-Mic19
pGEM4ZMic19G2A(1-20)-XhoI-(21-127)-KpnI-(128-170	Mic19 G2A(1-20)-LE-Mic19(21-127)-GT-Mic19(128-170)	SP6	pGEM4Z	Mic19(20)-XhoI-Mic19(21)-F<br>Mic19(127)-kpnI-Mic19(128)-R	N/A	pGEM4Z-Mic19G2A
pGEM4Z-XhoI-(21-127)-KpnI-(128-170)	Mic19 (21-127)-GT-Mic19(128-170)	SP6	pGEM4Z	met-XhoI-Mic19(21)-F<br>Mic19(127)-kpnI-Mic19(128)-R	N/A	pGEM4Z-Mic19Δ20
pGEM4Z-Mic19WT(1-20)-phoA(141-240)-(128-170)	Mic19(1-20)-LE-PhoA(141-240)-GT-Mic19(128-170)	SP6	pGEM4Z-Mic19WT(1-20)-xhoI-(21-127)-KpnI-(127-17	Mic19(20)-XhoI-PhoA(141-)-F<br>Mic19(127)-KpnI-PhoA(141-)-R	XhoI/KpnI	E.coli genomic DNA
pGEM4Z-Mic19G2A(1-20)-phoA(141-240)-(128-170)	Mic19 G2A(1-20)-LE-PhoA(141-240)-GT-Mic19(128-170)	SP6	pGEM4ZMic19G2A(1-20)-xhoI-(21-127)-KpnI-(127-17	Mic19(20)-XhoI-PhoA(141-)-F<br>Mic19(127)-KpnI-PhoA(141-)-R	XhoI/KpnI	E.coli genomic DNA
pGEM4Z-phoA(100)-(128-170)	PhoA(141-240)-Mic19(128-170)	SP6	pGEM4Z-xhoI-(21-127)-KpnI-(127-170)	Met-xhoI-phoA(141-)-F<br>Mic19(127)-KpnI-PhoA(141-)-R	XhoI/KpnI	E.coli genomic DNA
pGEM4Z-phoA(80)-(128-170)	PhoA(141-220)-Mic19(128-170)	SP6	pGEM4Z-phoA(100)-(127-170)	phoA(141-220)-F<br>SP6-R	N/A	pGEM4Z-phoA(140-220)-(127-170)
pGEM4Z-phoA(60)-(128-170)	PhoA(141-200)-Mic19(128-170)	SP6	pGEM4Z-phoA(100)-(127-170)	phoA(141-200)-F<br>SP6-R	N/A	pGEM4Z-phoA(140-220)-(127-170)
pGEM4Z-3×FLAG	3×FLAG	SP6	pGEM4Z	BamHI-3FLAG-F<br>SalI-3FLAG-R	BamHI/SalI	PFA6a-3×FLAG
pGEM4Z-Mic19WT-3×FLAG	Mic19-3×FLAG	SP6	pGEM4Z-3×FLAG pGEM4Z	SacI-Mic19(-50)-F<br>BamhI-Mic19non-R	SacI/BamHI	pGEM4Z-Mic19
pGEM4Z-Mic19G2A-3×FLAG	Mic19 G2A-3×FLAG	SP6	pGEM4Z-3×FLAG pGEM4Z	SacI-Mic19(-50)-F<br>BamhI-Mic19non-R	SacI/BamHI	pGEM4Z-Mic19G2A
pGEM4Z-Mic19Δ20-3×FLAG	Mic19 Δ20-3×FLAG	SP6	pGEM4Z-3×FLAG pGEM4Z	SacI-Mic19(-50)-F<br>BamHI-Mic19non-R	SacI/BamHI	pGEM4Z-Mic19Δ20
pGEM4Z-Mic19C146S/C157S-3×FLAG	Mic19 C146S/C157S-3×FLAG	SP6	pGEM4Z-3×FLAG pGEM4Z	SacI-Mic19(-50)-F<br>BamHI-Mic19non-R	SacI/BamHI	pGEM4Z-Mic19C146S/C157S
pGEM4Z-Mic19G2A/C146S/C157S-3×FLAG	Mic19 G2A/C146S/C157S-3×FLAG	SP6	pGEM4Z-3×FLAG pGEM4Z	SacI-Mic19(-50)-F<br>BamHI-Mic19non-R	SacI/BamHI	pGEM4Z-Mic19G2A/C146S/C157S
pGEM4Z-pSu9-DHFR	Su9-DHFR	SP6	N/A	N/A	N/A	Kanamori *et al.,* 1999
pGEM4Z-Tim23	Tim23	SP6	N/A	N/A	N/A	N/A
pGEM4Z-AAC	AAC	SP6	N/A	N/A	N/A	Tamura *et al.,* 2009
pRS314	N/A	N/A	N/A	N/A	N/A	N/A
pRS314-pro	N/A	*MIC19*	pRS314	SacI-mic19pro-F<br>xhoI-mic19pro-R	SacI/XhoI	yeast genomic DNA
pRS314-pro-ter	N/A	*MIC19*	pRS314-pro	XhoI-mic19ter-F<br>KpnI-mic19ter-R	XhoI/KpnI	yeast genomic DNA
pRS314-Mic19WT-FLAG	Mic19-3×FLAG	*MIC19*	pRS314-pro-ter	XhoI-mic19(-50)FLAG-F<br>XhoI-mic19FLAG-R	XhoI	pGEM4Z-Mic19WT-3×FLAG
pRS314-Mic19G2A-FLAG	Mic19 G2A-3×FLAG	*MIC19*	pRS314-pro-ter	XhoI-mic19(-50)FLAG-F<br>XhoI-mic19FLAG-R	XhoI	pGEM4Z-Mic19G2A-3×FLAG
pRS314-Mic19Δ20-FLAG	Mic19 Δ20-3×FLAG	*MIC19*	pRS314-pro-ter	XhoI-mic19(-50)FLAG-F<br>XhoI-mic19FLAG-R	XhoI	pGEM4Z-Mic19Δ20-3×FLAG
pRS314-Mic19C146S/C157S-FLAG	Mic19 C146S/C157S-3×FLAG	*MIC19*	pRS314-pro-ter	XhoI-mic19(-50)FLAG-F<br>XhoI-mic19FLAG-R	XhoI	pGEM4Z-Mic19C146S/C157S-3×FLAG
pRS314-Mic19G2A/C146S/C157S-FLAG	Mic19 G2A/C146S/C157S-3×FLAG	*MIC19*	pRS314-pro-ter	XhoI-mic19(-50)FLAG-F<br>XhoI-mic19FLAG-R	XhoI	pGEM4Z-Mic19G2A/C146S/C157S-3×FLAG
pNHK53	OsTIR1-9xMYC	*ADH1*	N/A	N/A	N/A	Nishimura *et al.,* 2009
pKan-AID*-9xMYC			N/A	N/A	N/A	Morawska *et al.* , 2013
pMK151			N/A	N/A	N/A	Kubota *et al* ., 2013

Table S1C. Primer list

Primers for plasmid constructions	
**Primer name**	**Sequence(5'→3')**
EcoRI-Mic10(-50)-F	TCCGAATTCGGCAGATTAACTTTGCTACGAG
BamHI-Mic10-stop-R	TCCGGATCCCTAAACCTTCGAGGATCTGAGG
EcoRI-Mic12(-50)-F	TCCGAATTCGGGGACAGAACAGACAGTGG
BamHI-Mic12-stop-R	TCCGGATCCCTAATTCTTACCCCAGGAGT
SacI-Mic19(-50)-F	TTCGAGCTCAAATTCAAGAACAAAAACAA
BamHI-Mic19-stop-R	TCCGGATCCTCAACCCATAACGAGCTTCT
EcoRI-Mic27(-10)-F	TTTGCACTCGAGAATTCAGCTTCCAAAATGGTAAATTTTTATG
SalI-Mic27-stop-R	CGGCCGCCCGGGTCGACTCATGCTTGCTCCAACTTTTC
EcoRI-Mic26(-50)-F	TCCGAATTCCGACTATAGCGCTCAAGGAT
BamHI-Mic26-stop-R	TCCGGATCCTCACTTCACGTTAACGCCAG
EcoRI-Mic60-F	TTTGCACTCGAGAATTCATGCTAAGAACTACTG
SalI-Mic60-stop-R	CGGCCGCCCGGGTCGACTCACAACGTCCTTATTTC
Mic19-C146S-F	GCAAAATTGACTGAGTCTCTTTTGGCCAATAAGGGC
Mic19-C146S-R	CGTTTTAACTGACTCAGAGAAAACCGGTTATTCCCG
Mic19-C157S-F	GGGCAAGCCATTGAATTCTTACGAAGAGATGGAAG
Mic19-C157S-R	CCCGTTCGGTAACTTAAGAATGCTTCTCTACCTT
Mic19-G2A-F	CTAATACAGAGTCAATCATGGCTTCAAACACTTCCAAAG
Mic19-G2A-R	GATTATGTCTCAGTTAGTACCGAAGTTTGTGAAGGTTTC
Mic19(Δ20)-F	CTAATACAGAGTCAATCATGCTAACACAGATCGATTTTTC
SP6-R	CCCAGGCTTTACACTTTATGCTTCCGG
4Z-BamHI-Mic19(-50)-F	CGGTACCCGGGGATCCAAATTCAAGAACAAAAAC
Mic19(-20)-XhoI-DHFR-R	ATGGTCGAACCTCGAGCGGAGTATAGACTTGTTGTTTTTC
pTNT-EcoRI-Tim9-F	TTTGCACTCGAGAATTCATGGACGCATTGAACTCCAAAG
pTNT-SalI-Tim9-R	CGGCCGCCCGGGTCGACTTATCGGCCCAAGCCTTGTCCC
EcoRI-Mic19WT(1-)-F	TTTGCACTCGAGAATTCATGGGTTCAAACACTTCCAAAG
EcoRI-Mic19G2A(1-)-F	TTTGCACTCGAGAATTCATGGCTTCAAACACTTCCAAAG
EcoRI-Mic19(-20)-R	ATGCGTCCATGAATTCCGGAGTATAGACTTGTTGTTTTTC
EcoRI-Mic19(-127)-R	ATGCGTCCATGAATTCTTTACCTTTGGCACCCCCATATTTC
EcoRI-Mic19(21-)-F	TTTGCACTCGAGAATTCATGCTAACACAGATCGATTTTTC
XhoI-Mic19WT(1-)-F	TCTTTTTGCACTCGAGATGGGTTCAAACACTTCCAAAG
XhoI-Mic19G2A(1-)-F	TCTTTTTGCACTCGAGATGGCTTCAAACACTTCCAAAG
XhoI-Mic19(21-)-F	TCTTTTTGCACTCGAGATGCTAACACAGATCGATTTTTC
XhoI-Mic19(-127)-R	TTTCAGTCATCTCGAGTTTACCTTTGGCACCCCCATATTTC
EcoRI-Mdm35-F	TTTGCACTCGAGAATTCATGGGGAATATAATGTCAGC
SalI-Mdm35-R	CGGCCGCCCGGGTCGACTCATTTGTCAACTTCTTTTAGTTTG
Mdm35G2A-F	CTCGAGAATTCATGGCGAATATAATGTCAGC
Mic19(20)-XhoI-Mic19(21)-F	CAAGTCTATACTCCGCTCGAGCTAACACAGATCG
Mic19(127)-KpnI-Mic19(128)-R	TTTTATCAGAGGTACCTTTACCTTTGGCACC
met-XhoI-Mic19(21)-F	CAGAGTCAATCATGCTCGAGCTAACACAGATCG
Mic19(20)-XhoI-PhoA(141-)-F	CTATACTCCGCTCGAGGCGCTGGGCGTCGATATTC
Mic19(127)-KpnI-PhoA(141-)-R	CCCGGTACCACCAGCGGTTGCCGTTTCAGC
Met-xhoI-phoA(141-)-F	GTCAATCATGCTCGAGGCGCTGGGCGTCGATATTC
phoA(141-220)-F	CAGCTGCTTAACGCTTCTGATAAAAAAACC
phoA(141-200)-F	GACCAGTGAAAAATGTTCTGATAAAAAAACC
BamHI-3FLAG-F	GCCGGATCCGACTACAAAGACCATG
SalI-3FLAG-R	GCCGTCGACTCACTTATCATCATCATCC
BamHI-Mic19non-R	GCCGGATCCACCCATAACGAGCTTC
SacI-mic19pro-F	CCCGAGCTCAAATTAGCAAAAAAAGGC
XhoI-mic19pro-R	CCCCTCGAGTTTTTTGATTAGCAATCTC
XhoI-mic19ter-F	AATCAAAAAAACTCGAGATAAGAAAAAGTATACATAAACTCGAC
KpnI-mic19ter-R	ACAAAAGCTGGGTACCATGGTACAAATTTAATGATGATAAAG
XhoI-mic19(-50)FLAG-F	AATCAAAAAAACTCGAGAAATTCAAGAACAAAAAC
XhoI-mic19FLAG-R	TTTTTCTTATCTCGAGTCACTTATCATCATCATCC
**Primers for deletion**	
**Primer name**	**Sequence(5'→3')**
Mic19-deletion-F	ACAAAAACAAGGTGGTATATCGACTAATACAGAGTCAATCGTTGTAAAACGACGGCCAGT
Mic19-deletion-R	CGAATTCTTTTTGGTCGAGTTTATGTATACTTTTTCTTATCACAGGAAACAGCTATGACC
**Primers for C-terminal AID tagging**	
**Primer name**	**Sequence(5'→3')**
Tom20AID F	GAAGCAAGGCCGAATCTGATGCGGTTGCTGAAGCTAACGATATCGATGACCGTACGCTGCAGGTCGAC
Tom20AID R	AGTAAAAGAAACAAAAACGGAGAAAAAAAGCAAGCAAAATGTTACTCTCAATCGATGAATTCGAGCTCG
**Primers for C-terminal FLAG tagging**	
**Primer name**	**Sequence(5'→3')**
TIM40-tag-F	GCAGAGCAATCAGATGTGAAAAAAGAACCATTGAATGAGGAATCCAAACCTCGGATCCCCGGGTTAATTAA
TIM40-tag-R	GTATCATCCTTTATACTTTTATACTTTTTACGTTCGGGAGATTTTCAGGAGATTGAATTCGAGCTCGTTTAAAC
